# Effect of ecological momentary assessment, goal-setting and personalized phone-calls on adherence to interval walking training using the InterWalk application among patients with type 2 diabetes—A pilot randomized controlled trial

**DOI:** 10.1371/journal.pone.0208181

**Published:** 2019-01-10

**Authors:** Laura Staun Valentiner, Ida Kær Thorsen, Malte Bue Kongstad, Cecilie Fau Brinkløv, Rasmus Tolstrup Larsen, Kristian Karstoft, Jens Steen Nielsen, Bente Klarlund Pedersen, Henning Langberg, Mathias Ried-Larsen

**Affiliations:** 1 CopenRehab, Department of Public Health, Section of Social Medicine, University of Copenhagen, Copenhagen, Denmark; 2 The Centre of Inflammation and Metabolism and the Centre for Physical Activity Research, Rigshospitalet, University of Copenhagen, Copenhagen, Denmark; 3 Centre of Inflammation and Metabolism, Department of Infectious Diseases, Rigshospitalet, University of Copenhagen, Copenhagen, Denmark; 4 Department of Clinical Pharmacology, Bispebjerg and Frederiksberg University Hospital, Copenhagen, Denmark; 5 Department of Endocrinology, Odense University Hospital, Odense, Denmark; 6 OPEN, Odense Patient Data Explorative Network, Odense University Hospital, Odense, Denmark; 7 The Danish Diabetes Academy, Odense University Hospital, Odense, Denmark; Victoria University, AUSTRALIA

## Abstract

**Objectives:**

The objective was to investigate the feasibility and usability of electronic momentary assessment, goal-setting and personalized phone-calls on adherence to a 12-week self-conducted interval walking training (IWT) program, delivered by the InterWalk smartphone among patients with type 2 diabetes (T2D).

**Methods:**

In a two-arm pilot randomized controlled trial (Denmark, March 2014 to February 2015), patients with T2D (18–80 years with a Body Mass Index of 18 and 40 kg/m2) were randomly allocated to 12 weeks of IWT with (experimental) or without additional support (control). The primary outcome was the difference between groups in accumulated time of interval walking training across 12 weeks. All patients were encouraged to use the InterWalk application to perform IWT for ≥90 minute/week. Patients in the experimental group made individual goals regarding lifestyle change. Once a week inquiries about exercise adherence was made using an ecological momentary assessment (EMA). In case of consistent self-reported non-adherence, the patients would receive a phone-call inquiring about the reason for non-adherence. The control group did not receive additional support. Information about training adherence was assessed objectively. Usability of the EMA was assessed based on response rates and self-reported satisfaction after 12-weeks.

**Results:**

Thirty-seven patients with T2D (66 years, 65% female, hemoglobin 1Ac 50.3 mmol/mol) where included (n = 18 and n = 19 in experimental and control group, respectively). The retention rate was 83%. The experimental group accumulated [95%CI] 345 [-7, 698] minutes of IWT more than the control group. The response rate for the text-messages was 83% (68% for males and 90% for females). Forty-one percent of the experimental and 25% of the control group were very satisfied with their participation.

**Conclusion:**

The combination inquiry about adherence using EMA, goal-setting with the possibility of follow-up phone calls are considered feasible interventions to attain training adherence when using the InterWalk app during a 12-week period in patients with T2D. Some uncertainty about the effect size of adherence remains.

**Trial registration:**

Clinicaltrials.gov NCT02089477

## Introduction

Physical activity (PA) is a cornerstone in the first line treatment of patients with type 2 diabetes (T2D) [1,2,3]. However, adherence to recommended levels of PA among patients with T2D is low [4,5].

Supervised exercise interventions can improve glycemic control [6], physical fitness [7] and quality of life [8] as well as reduce cardiovascular risk factors [8–10]. Yet, whereas supervised exercise is superior to non-supervised exercise in improving glycemic control in patients with T2D, advice-based exercise therapy is not [6,10]. This emphasizes the importance of supervision in successful implementation of exercise therapy in clinical care of T2D. However, challenges to supervised exercise include cost and time restrictions in treatment delivery. This creates challenges, especially given increasing T2D prevalence [11]. Moreover, previous studies have established several internal and external barriers for sustaining behavior change with PA in patients with T2D. These barriers can be demographic, social and psychological factors including poor physical fitness, lack of self-efficacy and lack of social support, lack of earlier experiences with exercise as well as logistic and contextual challenges [12–17]. Consequently, there is a need for approaches promoting PA for the T2D population that do not rely on direct exercise supervision.

Electronic health (eHealth) solutions involving communication technologies such as computers, telephones and videos are already recognized as potential tools to overcome some of the identified external barriers and improve adherence to various components of the clinical care of T2D [18–23]. However, several studies emphasize that technology-based interventions are more effective if combined with additional adherence supportive components [24,25]. In this regard, automated electronic inquiries about adherence have been found to be effective in improving diabetes self-management with a significant effect on glycemic control [20] and health behavior [26,27]. Evidence also suggest that automated electronic inquiries about adherence can be used to enhance PA among patients with T2D [28]. Moreover, previous research suggests that goal-setting is an effective tool to overcome several of the internal barriers for patients with T2D when it comes to changing behavior [29,30]. Based on these findings, we have developed a structured intervention using goal-setting, ecological momentary assessment (EMA) of adherence to PA, and the possibility for follow-up phone calls. The intervention aims at attaining adherence to a mobile-based application (the InterWalk app) which delivers an interval walking training (IWT) program [31].

The primary aim of this 12-week pilot randomized controlled trial was to investigate feasibility of inquiries about adherence to interval walking training using EMA, goal-setting and phone calls as interventions to attain adherence to an IWT intervention delivered by the smartphone application InterWalk in patients with T2D. Secondarily, we aimed to investigate usability of EMA and the effect on changes in self-reported PA, health related quality, aerobic capacity, glycated hemoglobin (HbA1c) and body composition from baseline to 12-week.

## Methods

### Design

**Fig 1:**
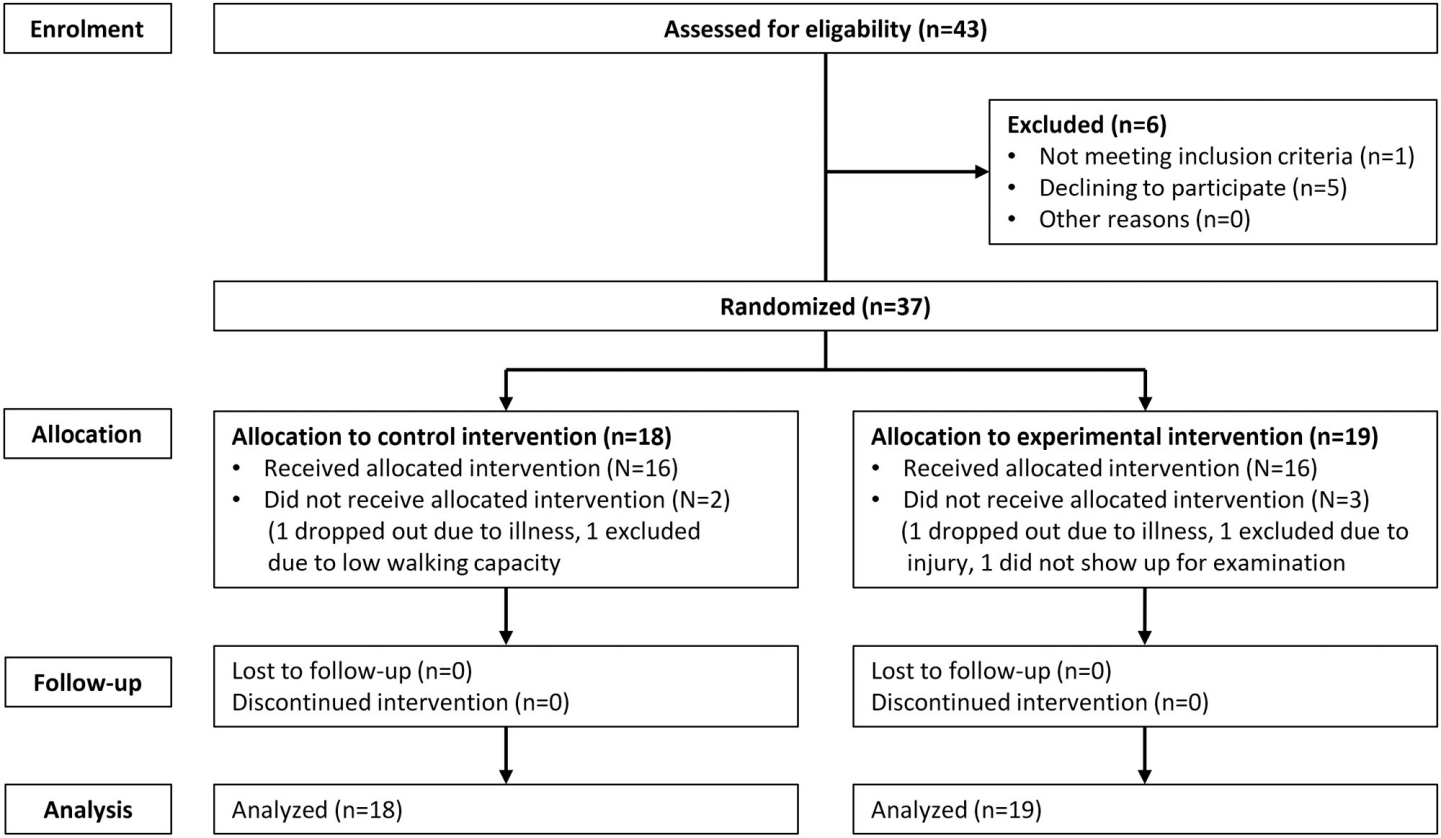
Flow chart. Pilot trial with randomization.

This trial was a single-center 12-week, two-arm pilot randomized controlled trial (RCT) [32], conducted at Copenhagen University Hospital, Rigshospitalet, March 12^th^ 2014 to February 12^th^ 2015. The protocol was approved by the *Scientific Ethical Committee* at the Capital Region of Denmark (H-1-2013-116, S1 and S2 Files), and the trial protocol was registered at *Clinicaltrials*.*gov* (NCT02089477). The study was conducted in accordance with the Helsinki Declaration [33]. Results from the trial are reported in line with the *CONsolidated Standards of Reporting Trials guidelines*: *extension to randomised pilot and feasibility trials* (CONSORT guidelines, S3 File) [34]. Other data from this study have previously been published [35,36], but no data on the primary outcome, usability or feasibility reported in this report was disclosed, nor were the group allocations. Flow of participants is presented in Fig 1.

### Patients and eligibility

Patients were recruited through; 1) an outpatient clinic for patients with T2D at the University Hospital of Copenhagen in the Capital Region of Denmark (Rigshospitalet), 2) information-meetings arranged by the *Danish Diabetes Association* in different cities in Capitol Region of Denmark, and 3) recruitment bulletins. Possible participants were considered eligible for inclusion if they had; 1) a diagnosis of T2D confirmed by their general practitioner, 2) a Body Mass Index (BMI) between 18 and 40 kg/m^2^ and 3) were between 30 and 80 years of age. The exclusion criteria were; 1) pregnancy, 2) smoking, 3) prescription of insulin analogues, 4) evidence of thyroid, liver, lung, heart or kidney disease, and/or restrictions to increased levels of PA [37]. Patients had a minimum of 24 hours to consider participation. Oral and written informed consent was obtained from patients prior to initiation of any trial procedures.

### Randomization and Blinding

Patients were randomly allocated in blocks of four into two groups using a computer-generated sequence (www.randomization.com). The two groups were; 1) IWT using the InterWalk app with additional support (experimental group), or 2) IWT using the InterWalk app without additional support (control group). The sequence was concealed in a locked cabinet at a location different from the test facility. All patients that were included and scheduled for baseline measurements were assigned consecutive numbers and allocated by a researcher (LSV), who was not involved with testing procedures. Blinding of research staff and patients was not possible following the allocation due to practical reasons, such as the ongoing communication between patients and the research staff across the intervention period.

### Interventions

**Fig 2:**
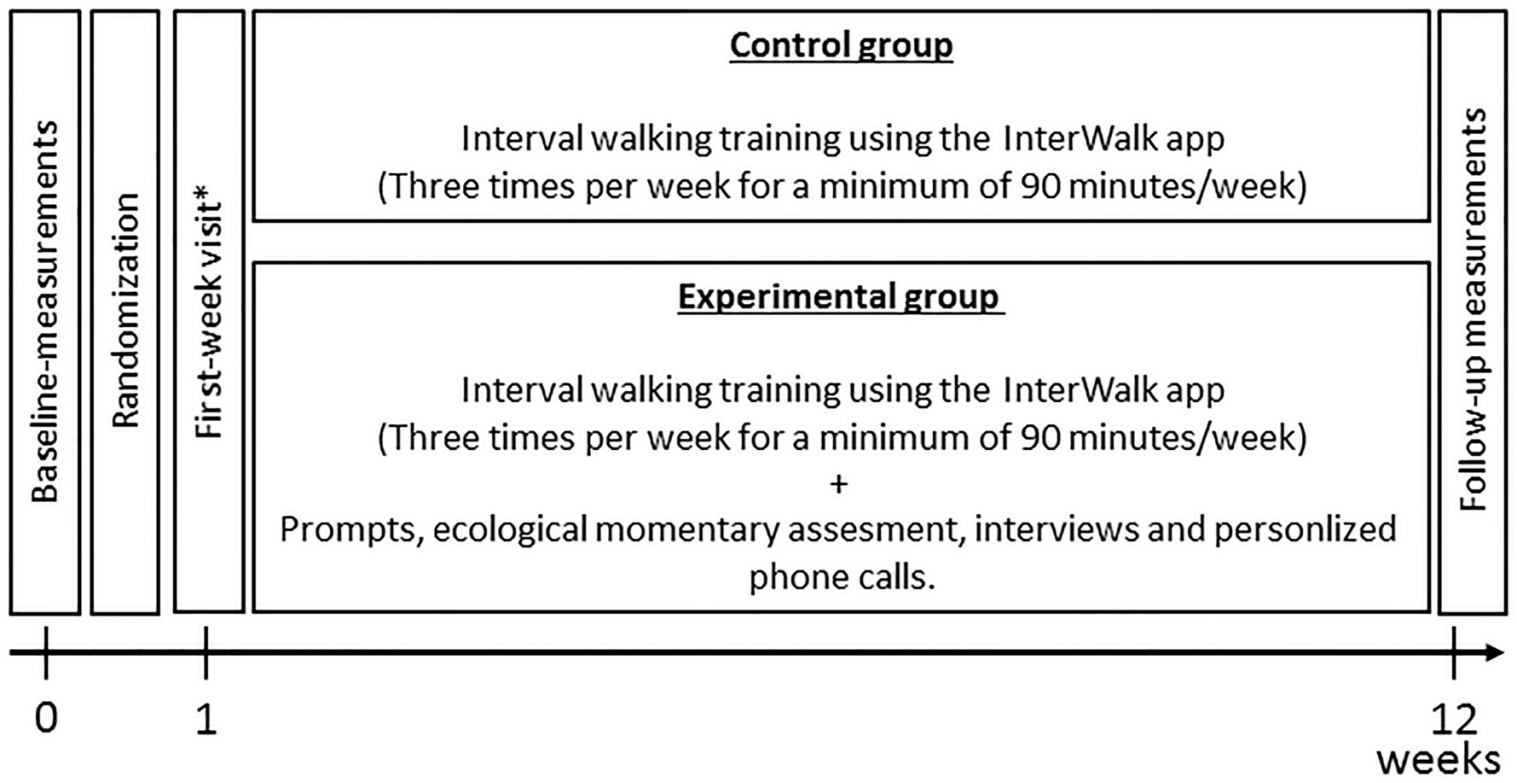
Timeline and overview of the interventions in the pilot trial. *1-week revisit: both the experimental and the control group had a 1-week revisit to ensure that they understood how to use the InterWalk app.

Following group allocation, all patients received a comprehensive introduction to the InterWalk app and walking test procedures (see below) [35]. All patients were encouraged to use the InterWalk app to perform ≥ 3 sessions of IWT per week for ≥ 90 minutes/week across 12 weeks. IWT was performed by the patients in free-living at home. All patients (N = 37) were asked to revisit the clinic one week following baseline measurements to ensure correct use of the app and to repeat the inbuild fitness test. Moreover, patients could contact researchers by e-mail or telephone during the intervention-period for technical support. Throughout the 12-week trial-period, the experimental group moreover received automated structured text-messages on a weekly basis as well as help setting goals concerning lifestyle change (see below). A group-walking component originally included in the intervention protocol but this component was left out due to time-constraints for the participants to re-visit the hospital facilities and the geographical scatter of the patients. This was decided prior to the implementation of the intervention. An overview of the pilot trial is illustrated in Fig 2.

#### Interval walking training (IWT)

IWT consisted of repetitive walking cycles alternating between 3 minutes fast and 3 minutes slow walking. Audio feedback through earphones on walking speed was continuously and automatically provided to the participant. The development of and a detailed description of the InterWalk app has been published elsewhere [31].

During baseline measurements, patients conducted a 7-minute standardized InterWalk Fitness Test (IWFT) to individualize walking intensity during IWT [35]. The walking test is comprised of four walking paces; 1^st^) a slow walking pace for 2 minutes, 2^nd^) 2 minutes of an intermediate pace, 3^rd^) 2 minutes of fast walking and 4^th^) 1 minute of very fast walking. Walking paces, and thus, walking intensities were defined by the individual participant. All walking tests were performed outside to reflect the patient’s home environment; i.e. a sidewalk with traffic noise. Following baseline measurements, all patients were instructed to perform ≥ 90 minutes of IWT the following week. Seven days after baseline measurements all patients were invited to revisit the test facility where they completed a new IWFT. The IWT instructions were repeated and patients were encouraged to ask any questions regarding usage of the InterWalk app. Written standardized working manuals for testing procedures, introduction to the InterWalk app and an interview-guide (in Danish–available upon request) were used to standardize procedures.

#### Experimental group

Inquiries about adherence to interval walking training using EMA, prompts and phone calls.

**Fig 3:**
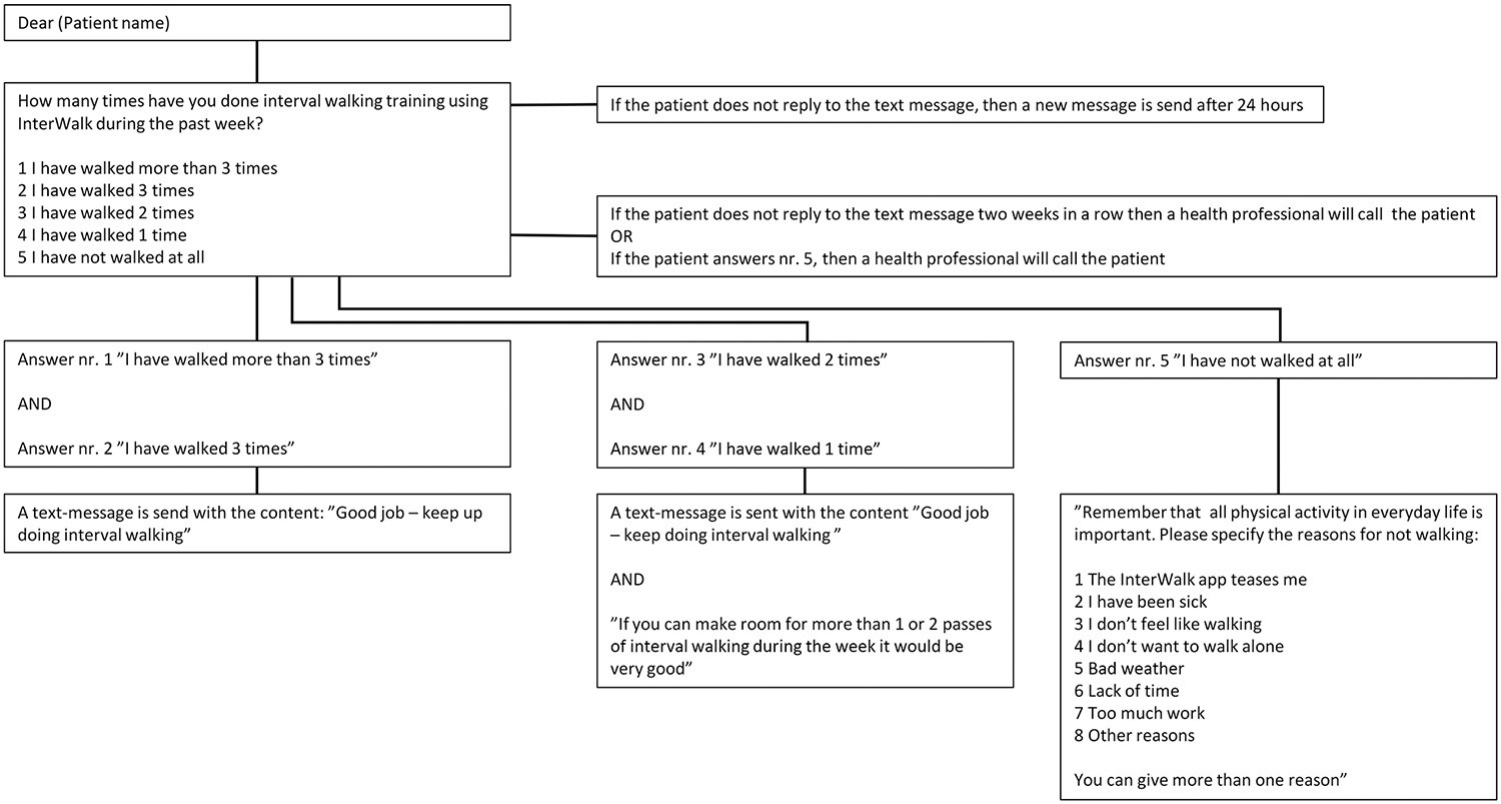
Weekly ecological momentary assessments (EMA’s) regarding frequency of training during the past week (all text has been translated from Danish to English language).

**Fig 4:**
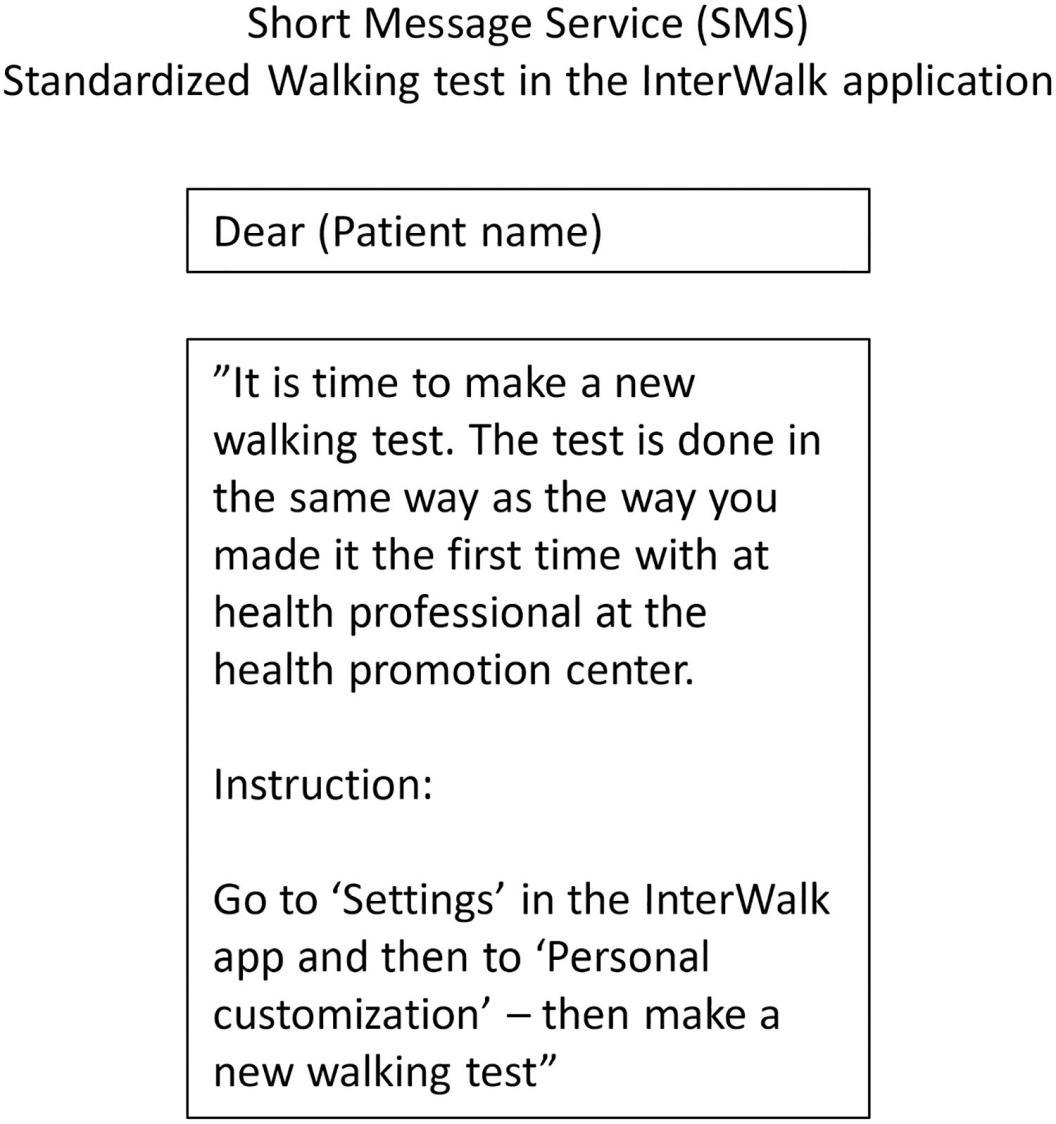
Prompts send every fourth week regarding the IWFT (the standardized walking test in the InterWalk application) (all text has been translated from Danish to English language).

Patients in the experimental group received prompts (one-way communication) and bi-directional (two-way communication) automated surveys (electronic momentary assessments) once per week throughout the 12-week trial-period using an automated short message service not in-cooperated in the InterWalk app (www.sms-track.dk). These EMA’s consisted of two different types of surveys which were sent automatically in the afternoon on Sundays; 1) a bi-directional weekly and interactive survey with inquires about the frequency of IWT during the past week (Fig 3) and 2) uni-directional prompts sent four weeks apart, encouraging patients to repeat the IWFT (Fig 4).

Patients were asked to respond to the weekly survey by choosing one of three answer-options provided (Fig 3). No reply or an answer indicating no performance of IWT for two consecutive weeks (Fig 3, answer number 5: “I have not walked at all”) resulted in a personal phone call from LSV. The conversation in this phone call was based on a semi-structured interview-guide to help patients reflect upon barriers to IWT. The semi-structured interview-guide consisted of two overall questions; 1) Do you experience barriers towards IWT if yes, which ones? 2) How can I help you to overcome these barriers?

Goal-setting

Following baseline assessments, the patients in the experimental group engaged in a preliminary interview [38] with individual goal-setting [39]. Patients set 2–3 functional goals related to their everyday life. The aim of the interview was to help the patients prioritize IWT in everyday life during the intervention period. The interview lasted 30 minutes and was conducted by two researchers (LSV or CFB). It took place following the allocation and prior to initiating IWT. In the interview, patients defined individual goals for the intervention-period using the S.M.A.R.T-principle (Smart, Measurable, Acceptable, Realistic goals within a given Timeframe) building on theory of goal-setting [39]. As such, goals were set according to goal-relevant activities, knowing that this can have an effect on performance and the meaningfulness of the goal to the individual patient [40]. Goals were recorded in the patient journal and each patient was provided a copy. The goals were not re-evaluated as otherwise stated in the trial registration and original protocol, however an automated prompt (uni-directional) to renew the goals were send every 4^th^ week.

### Outcomes and measurements

The primary outcome was adherence to IWT measured as total accumulated minutes of IWT during the 12-week intervention using data from the InterWalk app. Secondary outcomes were included *post hoc* and they were based on a concurrent qualitative investigation (*unpublished data*) with persons with T2D attending a rehabilitation program. This investigation guided the inclusion of the following measures as secondary outcomes; usability of structured text-messages, self-reported PA, satisfaction with trial participation, health related quality of life, changes in aerobic capacity, glycated hemoglobin (HbA1c) and anthropometric measures as secondary outcomes. As such, these outcomes were not described in the protocol or the trial registration, they are therefore considered exploratory. The outcomes were assessed from baseline to 12-week follow-up measurement.

#### Adherence to interval walking training

All information regarding training intensity, IWT-data and data from the IWFT (information concerning walking test, training sessions, intensity and number of steps per day) was stored locally in the iPod or smartphone and automatically transferred to a central database at the *Danish Strategic Research Centre for Type 2 Diabetes* (DD2) upon connection to a mobile data network or Wi-Fi [31]. All data from the app was encrypted and access to data was not possible during the intervention period.

Self-reported adherence to IWT using the InterWalk app was based on two questions with three response options on a likert scale [41]. Questions were; *1) How many times per week did you use the InterWalk application to do interval walking training during the last 12 weeks*? (options were 1) 1–2 times per week, 2) 3 times per week or 3) more than 3 times per week), *2) When using the InterWalk application to perform interval walking training*, *for how long did you walk per session*? (options were 1) 10–15 minutes, 2)16-30 minutes or 3) more than 30 minutes).

Self-reported renewal of the IWFT across groups, was based on one question. The response was dichotomized with a yes/no option. The question was *Have you renewed the standardized InterWalk Fitness Test at 4 weeks/at 8 weeks*? These questions were given to all patients after 12 weeks.

#### Usability of structured the electronic momentary assessment

Data concerning usability of the EMA component was obtained through patient response rates and replies to the weekly EMA’s (Figs 3 and 4).

#### Satisfaction

Self-reported satisfaction with participation in the trial and prospective usage of the InterWalk app were obtained at the 12-week follow-up assessment. Satisfaction was based on two questions with the three response options [41]. They were: 1) *“Have you been satisfied with your participation in this 12-week trial*?*”* (Options were a) *“Yes very satisfied”*, b)”*Satisfied” or* c)*“Not satisfied”)* and 2) *“Will you use the InterWalk app to perform interval walking training after the 12-week trial period”* (Options were a) *“Yes*, *several times a week/yes”*, b) *“Once a week/yes*, *sometimes/no”*, c) *“I don’t think so/don’t know”*)?

#### Physical activity

Changes in physical activity level during the past 4 weeks was determined using the “Recent Physical Activity Questionnaire” (R-PAQ)[42]. The questionnaire covered the domains “PA at home” (domain A), “PA in relation to work and during recreation”(domain B) and “PA in relation to transport” (domain C). Self-reported physical activity energy expenditure (PAEE) was used to estimate volume of recent PA [43].

#### Quality of life

Health related quality of life was measured through the Short-Form Health Survey (SF-12) containing 12 items covering general health. The measure is divided into two higher-order summary measures; namely a Mental Health and a Physical Health component summary, each with four sub-scales. The Mental health summary measure is comprised of the scales; ‘vitality’, ‘social functioning’, ‘role-emotional’ and ‘mental health’. The Physical Health summary measure is comprised of the scales; ‘physical functioning’, ‘role-physical’, ‘bodily pain’ and ‘general health’ [44].

#### Aerobic capacity

Aerobic capacity was measured by a graded maximal oxygen consumption test conducted on a treadmill (Technogym Runrace, Gambettola, Italy). The test consisted of a 5-minute warm-up at a slow walking pace (determined by the patient) at 0% incline followed by 2-minute stages of increasing inclines of 2% per stage at brisk walking pace (determined by the patient). The test stopped when the following criteria were met: plateauing heart rate (monitored with Polar Electro, Holte, DK) and plateauing oxygen consumption (VO_2_) with incremental workloads and a respiratory exchange ratio >1.1 [45]. Oxygen consumption was assessed using indirect calorimetric measurements (CPET, Cosmed, Italy).

#### Glycemic control

Glycemic control, expressed as HbA1c, was analyzed using standard procedures at a central laboratory (Rigshospitalet, Denmark).

#### Anthropometry

Dual x-ray absorptiometry (DXA) scan (iDXA; Lunar, Prodigy Advance; GE Healthcare, Madison, WI) was used to assess Body composition (total fat-mass, android fat-mass and gynoid fat-mass). Information regarding height, weight and waist circumference were obtained using standard procedures.

### Statistical methods

A formal sample size calculation was not performed, given that the trial serves as a pilot trial [34]. The sample size was determined based on financial restraints; either at 50 patients or the number of participants enrolled by November 9^th^ 2014.

Due to the nature of the trial, it was decided not to perform any formal statistical efficacy analyses [34]. For the primary analysis, difference in means of total accumulated minutes of interval training with 95% confidence intervals (95% CI) between the allocated groups, was derived from a mixed effects model with fixed effect for group (2 levels), age (continuous) and sex (2 levels) and reported according to intention to treat (ITT). As all data was prospectively registered in an online database, and all data, therefore, were available (including data from patients lost to follow-up), no imputation procedures were performed. As a sensitivity analysis, we performed a complete-case analysis including patients who attended both baseline and 12-week follow-up measurements. We investigated assumptions of normal distributed residuals of the mixed effects model. In an explorative analysis, adherence was dichotomized (yes/no of meeting 90 minutes of IWT/week) and the absolute risk of adherence was reported as the risk difference (risk of adherence in the experimental group minus the risk of adherence in the control group). Mean difference and 95% CI from continuous secondary outcomes (changes from baseline to 12-week follow-up) were derived from mixed linear models with same fixed factors as for primary outcome, but additionally adjusted for the respective baseline values. Baseline characteristics, usability and participation satisfaction outcomes are reported as means with standard deviations, medians with interquartile ranges or numbers and proportions as appropriate. Statistical analyses were performed using STATA IC 13.1 (Stata 210 Corp, Texas, USA).

## Results

### Sample

Baseline characteristics are shown in Table 1. Forty-three patients were assessed for eligibility. Five patients declined participation (primarily due to no interest in participation) and one patient did not meet the inclusion criteria. Thus, 37 patients were randomly allocated to either experimental group (n = 19) or control group (n = 18). Thirty-two patients completed the intervention. Following the allocation, two patients withdrew from the control group: one due to general illness and one was not able to walk. Three patients from the experimental group withdrew: one due to general illness, one due to injury not related to the trial and one did not attend the baseline examination. Thirty-five patients (N_experimental_ = 19 and N_control_ = 16) completed the re-visit in the laboratory. In total 37 patients were included in the ITT analysis. The sample was predominantly overweight with slightly more females than males (65% vs. 35%) (Table 1).

**Table 1:** Baseline demographic characteristics on all randomized participants.

Characteristics	*Total*	*Control group (IWT only)*	*Experimental group (IWT + SMS-track)*
Number of participants (N)	37	18	19
Female, No (%)	24 (64.9)	11 (29.7)	13 (35.1))
Age (years)	65.9 (6.8)	65.1 (6.4)	66.7 (7.3)
Height (cm) ◆	167.4 (8.2)	167.0 (8.5)	167.7 (8.1)
Body mass (kg) ◆	82.5 (17.6)	83.3 (17.5)	81.7 (18.1)
HbA1c (mmol/mol)◆	50.3 (12.1)	51.6 (12.9)	49.1 (11.6)
Body mass index (kg/m^2^) ◆	29.4 (5.7)	29.8 (5.6)	29.0 (6.0)
Total fat mass (%) ◆	37.9 (8.7)	38.5 (8.0)	37.3 (9.5)
Android fat mass (%) ◆	45.9 (8.7)	46.9 (7.2)	45.0 (10.0)
Gynoid fat mass (%) ◆	40.1 (9.7)	39.8 (8.7)	40.4 (10.8)

^**◆**^ Height, Body mass index, HbA1C, Total fat mas, Android fat mass, and Gynoid fat mass are based on 35 patients. Data are in means (standard deviations) or numbers (%)

### Adherence to interval walking training

**Fig 5:**
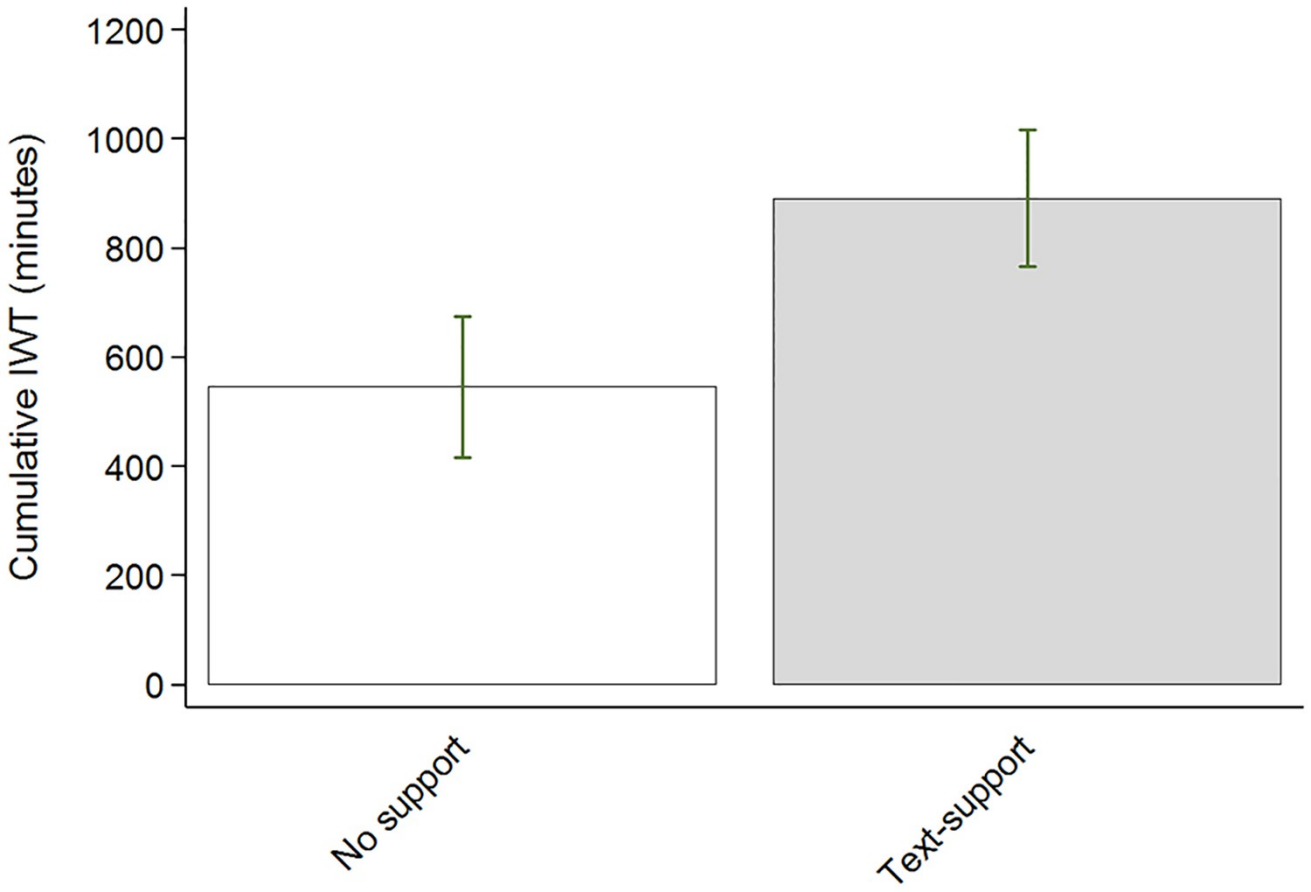
Accumulated minutes of interval walking training across 12-weeks by group. Data are means with standard error.

**Fig 6:**
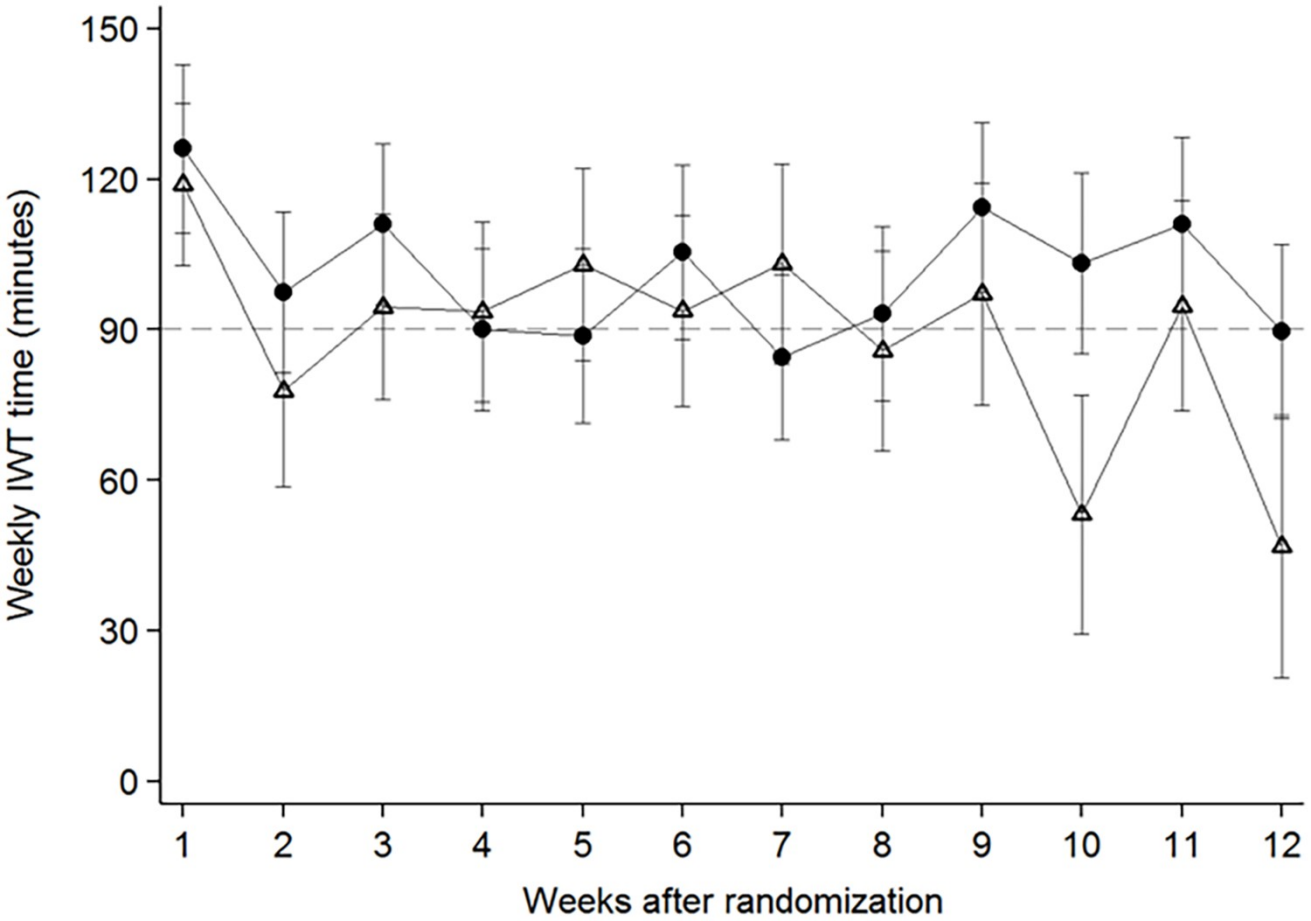
Weekly interval walking training minutes during week 1 through 12 of follow-up for the intention-to-treat population. Closed circles represent the experimental group and open triangles are the control group. Data are means and error bars represent standard error. IWT denotes Interval walking training.

The experimental group accumulated [95%CI] 345 [-7, 698] minutes of IWT more than the control group, equivalent to 29 minutes/week (Fig 5). Complete-case analysis (n = 32) showed that the experimental group accumulated [95%CI] of 434 [63, 804] minutes of IWT more than the control group, equivalent to 36 minutes/week. Risk difference [95%CI] of reaching the recommended level of IWT was 36% [9, 63], with the proportion of patients reaching recommended level of 90 minutes/week of IWT was 47% (9 out of 19) in the experimental group compared to 11% (2 out of 18) of patients in the control group. The weekly accumulated minutes of IWT per group is depicted in Fig 6. Self-reported adherence to IWT and renewal of IWTF are described in Table 2.

**Table 2:** Usability and feasibility of the interwalk app to do interval walking training.

	Control group (*n = 18*)	Experimental group (*n = 19*)	Total (*n = 37*)
Performing Interval walking training using the InterWalk app^1^			
*1–2 times/week*	4 (12.5)	1 (3.1)	5 (15.6)
*3 times/week*	7 (21.9)	8 (25.0)	15 (46.9)
*more than 3 times/week*	4 (12.5)	7 (21.9)	11 (34.4)
*Missing answer*	1 (3.1)	0 (0.0)	1 (3.1)
Trained minutes per session using the InterWalk app^2^			
*10–15 minutes*	0 (0.0)	1 (3.1)	1 (3.1)
*16–30 minutes*	6 (18.8)	3 (9.4)	9 (28.1)
*more than 30 minutes*	10 (31.3)	12 (37.5)	22 (68.8)
*missing answer*	0 (0.0)	0 (0.0)	0 (0.0)
Renewal of Standardised InterWalk Fitness Test (IWFT)^3^			
*4 weeks*			
*yes*	7 (21.9)	10 (31.3)	17 (53.1)
*no*	7 (21.9)	5 (15.6)	12 (37.5)
*Missing answer*	2 (6.3)	1 (3.1)	3 (9.4)
*8 weeks*			
*yes*	5 (15.6)	9 (28.1)	14 (43.8)
*no*	9 (28.1)	6 (18.8)	15 (46.9)
*Missing answer*	2 (6.3)	1 (3.1)	3 (9.4)

Data are n (%).

^1^How many times per week did you use the InterWalk during the last 12 weeks?

^2^When using InterWalk application to perform interval walking training, for how long did you walk per session?

^3^Have you renewed the standardised InterWalk Fitness Test at 4 weeks/at 8 weeks?

### Usability of electronic momentary assessment

The response rates to the EMA’s are reported in Table 3. More females than males responded to weekly EMA’s in the experimental group. A total of 30 follow-up questions on weekly EMA’s were sent with a response rate of 100% (Table 3). Three telephone calls (by LSV) to different patients were made (due to consistent reporting of non-adherence to IWT).

**Table 3:** Usability of ecological momentary assessment (experimental group only)*.

EXPERIMANTAL GROUP (n = 18)◆			
	Male	Female	Total
*Total replies*, *No (%)*^1^	49 (68)	130 (90)	179 (83)
*Total missing replies*, *No (%)*	23 (32)	14 (10)	37 (17)
*Total answered follow-up questions*^2^, *No (%)*	21 (43)	9 (7)	30 (100)
*Reported reasons for not using the InterWalk app*^3^	*No (%)*		
Illness	6(20)		
No motivation	1(3)		
Don’t want to walk alone	3(7)		
Bad weather	4(10)		
Lack of time	1(3)		
Due to work	1(3)		
Other reasons	14(53)#		

* Only Bi-directional text-messages

^◆^ One patient was never set up in the electronic momentary assessments systems due to never showing up for examination

^1^Once a week during the 12 weeks of intervention the patient was asked: “How many times did you use InterWalk during the last week?”

^2^If patients answered, that they did not use InterWalk during the last week, they were asked: “What is the primary cause to not using InterWalk?”

^3^Reported Reasons for not using InterWalk app stated in the follow-up question.

^#^ Other reasons stated include: Vacation, no InterWalk application and walked without using the InterWalk app.

### Satisfaction with participation and prospective usage of the InterWalk app

Thirteen patients (68%) were very satisfied, two patients (11%) were satisfied and one (5%) was not satisfied with their participation in the experimental group. The corresponding numbers in the control group were eight patients (44%), seven (39%) and one (6%). A total of 78% patients (experimental group; n = 14, control group; n = 11) reported that they intended to use the InterWalk app to perform IWT after the trial had ended. Nine patients (28%) in the experimental group and ten patients (31%) in the control group reported that they intended to use the app for IWT several times a week. Five (16%) patients in the experimental group reported that they would only use the InterWalk app once a week/once in a while compared to one patient in the control group.

### Other outcomes

Other self-reported and metabolic outcomes are reported in Table 4. Data does not indicate any between-group difference on the metabolic outcomes. However, the data indicates that mental well-being increases in response to the intervention.

**Table 4:** Within-group changes and between-group comparisons of changes in secondary outcomes from baseline to 12 weeks.

	*Control group (IWT only)*				*Experimental group*				*Between-group difference*		
	*N*	*Change*	*LCL* *95%*	*UCL* *95%*	*N*	*Change*	*LCL* *95%*	*UCL* *95%*	*Mean diff*.	*LCL* *95%*	*UCL* *95%*
Aerobic capacity (ml O_2_/min)^1^	14	94.54	-5.10	194.18	15	51.81	-44.35	147.97	-42.73	-183.25	97.79
Physical activity (kJ/kg/day)^1^	15	-3.64	-9.09	1.82	14	-1.14	-6.80	4.52	2.50	-5.54	10.54
Body composition^1^											
*Total fat mass (kg)*	15	-0.004	-0.01	0.004	16	-0.003	-0.009	0.004	0.001	-0.009	0.01
*Android fat mass (kg)*	15	-0.002	-0.01	0.007	16	-0.003	-0.009	0.008	0.002	-0.01	0.01
*Gynoid fat mass*	15	-0.66	-1.71	0.40	16	-0.001	-0.01	0.008	-0.0002	-0.01	0.01
*Body weight (kg)*^1^	15	-0.001	-0.01	0.008	16	-0.35	-1.38	0.67	0.39	-1.19	1.80
*HbA1c (mmol/mol)*^1^	15	-0.27	-2.00	1.45	16	1.57	-0.10	3.24	1.84	-0.59	4.28
Quality of life											
*Physical*	15	3.31	-0.62	7.24	12	1.82	-2.58	6.22	-1.49	-7.47	4.49
*Mental*	15	-3.72	-8.24	0.81	12	3.60	-2.00	8.12	6.78	-0.04	13.60

Data are mean change, difference in mean change, upper and lower 95% confidence limits (UCL95% and LCL95%),

^1^Adjusted for sex and baseline value

## Discussion

In this pilot trial, we assessed the feasibility and usability of a multi-component consisting of EMA’s, goal-setting and phone calls on adherence to IWT delivered by the InterWalk app in patients with T2D. The main finding was that the intervention increased the adherence to IWT, as assessed by time spent performing IWT. However, considerable imprecision around the effect estimate was observed. Moreover, adherence to IWT was similar between the groups until week eight of the intervention period. After that, the experimental group retained adherence, whereas adherence in the control group tended to decline. Patients in the experimental group found EMA’s highly satisfactory and had a high response rate to the surveys.

In line with our findings, Agboola et al. observed that daily personalized messages increased adherence to walking [28]. Likewise, Yom-Ton et al. observed that daily short messages (through SMS) with an in-built reinforcement learning algorithm using personalized feedback, could increase adherence to walking in a sedentary group of patients with T2D [46]. Arambepola et al. found that automated brief-messages targeting diet and PA via mobile devices produced a modest improvement on glycemic control [20]. And finally, Morton et al. found that a combination of tailored electronic messages, goal-setting and personalized feedback was more effective in enhancing PA, than text-messages alone in a population of people at high risk of T2D [47]. These findings are in agreement with previous findings [48–51]. Even though EMA’s in our trial did not have an in-built learning algorithm, patients were adherent to IWT. However, it can be speculated that adherence to IWT in the experimental group might have been higher if such an in-build learning algorithm had been incorporated.

In our trial, only three telephone calls to three different patients were made to inquire about changes in self-reported adherence during the intervention period. This has to be seen in relation to a total of 230 telephone calls that could potentially have been made. Although the number of calls made was low, the possibility of engaging a personal contact may be important. Yavuz et al. have reported better persistence and adherence to insulin treatment when electronic messages were supported by sequential telephone interviews in patients with T2D [52]. The resources required to monitor responses to EMA’s in our trial were low, indicating that monitoring treatment adherence with EMA’s and follow-up telephone interviews may a feasible primary care tool to help prevent relapse to a physically inactive lifestyle in patients with T2D. Moreover, this approach may be scalable due to low costs and no major strains on health care professionals. Our data suggest that males had a lower response rate to the electronic messages than women. Rather than investigating sex-specific differences, most studies report results on group level adjusted for sex in relation to ethnicity, level of health literacy and education, income or willingness to use electronic messages as a tool for better adherence [51,52,53]. In contrast to our results Yom-Tov reported that women were less likely to respond to electronic messages compared to men and that sex and reaction to messages were significantly correlated, indicating that feedback need to be tailored according to sex [46]. Our study was not designed to investigate sex-differences in usability of EMA’s, therefore this observation needs further attention in relation to tailoring text-messages in the best way when designing a confirmatory trial.

Interventions aimed at creating change PA behavior through such means, and EMA’s, have shown positive effects in relation to health-related behavior changes including PA [54–56]. This holds true both when the intervention is used as a co-intervention to PA [28,48] and as a self-management tool [49,50]. The observed response rates to text-messages in our trial were however, comparable to those found in similar studies using a theoretical framework when designing usability and acceptable electronic messages in health care [26,57–61]. However, working with a theoretical framework in the design phase of a confirmatory trial, may however help obtain even higher adherence to digitally supported exercise, when employing EMA’s as an intervention.

We did not observe any effect on metabolic risk factors, VO_2peak_ nor body composition, despite the seemingly higher adherence in the experimental group compared to the control group. Contrary to our findings, however, effects of IWT on these parameters have previously been reported in patients with T2D [45, 62] and healthy elderly persons [63–65]. Many patients failed to renew the IWFT in the app, indicating that these weekly prompts were not decisive to prompt patients to renew the IWFT. The intensity of IWT was personalized and aimed at 85% of VO_2peak_ based on the IWFT, which has been validated to patients with T2D by Brinkløv et al. [35]. However, the limits of agreement based on the IWFT were very wide. Therefore, the actual walking intensity during IWT may have been grossly underestimated compared to the intended intensity [6,66]. The lack of renewal, mentioned above, and possible underestimation of the actual exercise intensity during IWT may therefore have been insufficient to improve metabolic risk factors in this study. Moreover, previous findings suggest that the effect of exercise on HbA1c is dependent of the HbA1c, when entering the intervention (i.e., patients with higher HbA1c values at baseline would improve glycemic control more as compared to patients with lower HbA1c values at baseline) [66]. Participants in our trial entered the study with an HbA1c close to the diagnostic criteria for T2D, which may explain the lack of effect on HbA1c. Moreover, we suspect that recommended volume (90 min per week) or frequency (3 times per week) may have been insufficient to improve metabolic markers as Umpierre et al. have suggested an inverse dose response relationship between exercise frequency and reductions in HbA1c, with larger reductions at higher frequencies (i.e., more weekly exercise sessions) [6]. Karstoft et al. did not observe an effect of IWT on a reduction in HbA1c, but rather the IWT reduced hyperglycemia in patients with T2D [45] suggesting that IWT reduces hyperglycemia rather than mean blood glucose (as expressed as HbA1c) in these patients [62]. Finally, the lower bound of the 95% CI on the primary outcome in the complete-case analysis indicates that the IWT adherence may have been very low to modest and thus explain the lack of effect on the metabolic risk factors. One or all the above, may have precluded an effect of IWT on HbA1c and other metabolic risk factors. Thus, a future confirmatory trial based on IWT in patients with T2D needs to consider an accurate estimation of intensity of IWT and/or an enforcement of the renewal of the IWFT. Moreover, besides increasing the sample size to increase precision of the adherence estimate, the volume and/or frequency of IWT will likely have to be higher to obtain clinically relevant improvements on cardiovascular risk factors.

Some limitations of the trial need to be addressed. First, we did not assess patients’ health literacy or digital readiness. Low health literacy related to diabetes knowledge, self-efficacy and self-care behavior and glycemic control is common in patients with T2D [67–69]. Moreover, several studies have reported that health literacy is positively related to health promoting behavior as PA in Patients with T2D [70,71]. Due to reliance on digital solutions to promote PA (app and EMA’s), generalizability to primary care may be precluded if patients in primary differs in the level of digital readiness and/or health literacy [72–74]. However, Bergner et al. found that patients with limited health literacy were as likely to respond to bi-directional text-messages as patients with adequate health literacy [75,76]. Thus, possible differences in health literacy may not necessarily affect translation of our findings into the primary care of T2D. However, if participants in our study possessed better skills in handling digital solutions in relation to health compared with patients in primary care [70,71], the adherence to IWT in our study may be higher than would observed in a clinical care context. To fully appreciate how to translate our findings into primary care in a confirmatory trial, investigations of targeting different dimensions (e.g. user, task and health care context dimensions) of eHealth literacy and their effect on adherence to PA interventions are warranted [77]. Secondly, we relied on self-reported adherence to IWT through EMA’s, in the communication with experimental group. As self-reported PA is prone information bias [78], the frequency of telephone inquiries made in this study may have been higher had the decision to inquire been based on objective assessments of adherence. Christensen at al., have suggested that direct personal interaction and feedback may be essential for some patients with multi-morbidity to adhere to self-management of their disease [79]. The low frequency of telephone inquiries, due to information bias may thus have caused an underestimation of the need for direct personal contact with a health care professional. There may have been a greater need for direct personal communication than detected by the self-reporting, which may have resulted in an increased risk of loss to follow-up or non-adherence. Thirdly, qualitative semi-structured interviews with participating patients to formally investigate feasibility and usability were not included as a part of this pilot trial. Using this approach, we might have gained a more in-depth knowledge in relation to usability, acceptability and relevance of EMA’s in concert with the InterWalk app. These insights can be obtained through a nested pilot investigation as a part of a confirmatory trial.

In conclusion, our findings support the use of EMA’s, goal-setting and phone-calls as feasible interventions to attain adherence to IWT, promoted by the InterWalk app, during a 12-week intervention period in patients with T2D. However, some uncertainty about the effect size of adherence and InterWalk acceptability still exist. The EMA-based approach was feasible with a minimum of contacts with patients, and the patients also provided a high response rate suggesting that the approach is usable for this patient group. Conducting a confirmatory trial with rigorous study design and longer follow-up period will provide robust evidence of the effectiveness of supporting the InterWalk app-based intervention with EMA’s and goal-setting on IWT adherence in patients with T2D.

## Supporting information

S1 File Approved study protocol (Danish/English). (PDF)

S2 File Approved study protocol (English). (PDF)

S3 File CONSORT checklist. (PDF)

## References

[1] Colberg SR. Sigal RJ Fernhall B Regensteiner JG. Blissmer Bj Rubin RRR. et al Exercise and Type 2 Diabetes. Diabetes Care. 2010;33(12):2692–6. 10.2337/dc10-1548 21115771 PMC2992214

[2] Plotnikoff RC, Costigan S a., Karunamuni ND, Lubans DR Community-based physical activity interventions for treatment of type 2 diabetes: A systematic review with meta-analysis. Front Endocrinol (Lausanne). 2013;4(JAN):1–17. 23372566 10.3389/fendo.2013.00003 PMC3557414

[3] American Diabetes Association (ADA). Standards of medical care in diabetes—2017. Diabetes Care. 2017;40 (sup 1)(1):s4–128.27979887

[4] Shultz JA, Sprague MA, Branen LJ, Lambeth S. A comparison of views of Individuals with Type 2 Diabetes Mellitus and Diabetes Educators about Barriers to Diet and Exercise. J Health Commun. 2001;6(2):99–115. 10.1080/10810730116985 11405082

[5] Unick JL, Gaussoin SA, Hill JO, Jakicic JM, Bond DS, Hellgren M, et al Objectively Assessed Physical Activity and Weight Loss Maintenance among Individuals Enrolled in a Lifestyle Intervention. Obesity [Internet]. 2017;0(0):1–7. Available from: http://doi.wiley.com/10.1002/oby.21971 10.1002/oby.21971 PMC5695666 28940967

[6] Umpierre D, Kramer CK, Leita CB, Gross JL, Ribeiro JP, Schaan BD. Physical Activity Advice Only or Structured Exercise Training and Association With HbA1c Levels in Type 2 Diabetes—A Systematic Review and Meta-analysis. JAMA. 2011;305(17):1790–9. 10.1001/jama.2011.576 21540423

[7] Balducci S, Zanuso S, Cardelli P, Salvi L. Changes in physical fitness predict improvements in modifiable cardiovascular risk factors independently of body weight loss in subjects with type 2 diabetes. Diabetes Care [Internet]. 2012;35:1347–54. Available from: http://www.wsjsw.gov.cn:8089/gate/big5/care.diabetesjournals.org/content/35/6/1347.full 10.2337/dc11-1859 22399699 PMC3357233

[8] Nicolucci A, Balducci S, Cardelli P, Zanuso S, Pugliese G. Italian Diabetes Exercise Study (IDES) Invesigators, Improvement of quality of life with supervised exercise training in subjects with type 2 diabetes mellitus. Arch Intern Med. 2010;171((21)):1951–3. 10.1001/archinternmed.2011.561 22123809

[9] Balducci S, Zanuso S, Nicolucci A, De Feo P, Cavallo S, Cardelli P, et al Effect of an Intensive Exercise Intervention Strategy on Modifiable Cardiovascular Risk Factors in Subjects With Type 2 Diabetes Mellitus. Arch Intern Med. 2010;170(20):1794–803. 10.1001/archinternmed.2010.380 21059972

[10] Avery L, Flynn D, Van Wersch A, Sniehotta FF, Trenell MI. Changing physical activity behavior in type 2 diabetes: A systematic review and meta-analysis of behavioral interventions. Diabetes Care. 2012;35(12):2681–9. 10.2337/dc11-2452 23173137 PMC3507564

[11] International Diabetes Federation. IDF DIABETES ATLAS 2015, Seventh Edition.

[12] Delamater AM. Improving patient adherence. Clin Diabetes [Internet]. 2006;24(2):71–7. Available from: http://cgi/reprint/24/2/71

[13] Kerkelä ES, Jonsson L, Lindwall M, Strand J. Individual experiences following a 6-month exercise intervention: A qualitative study. Int J Qual Stud Health Well-being. 2015;10:1–12. 10.3402/qhw.v10.26376 PMC4539384 26282865

[14] Lidegaard LP, Schwennesen N, Willaing I, Faerch K. Barriers to and motivators for physical activity among people with Type 2 diabetes: patients’ perspectives. Diabet Med [Internet]. 2016;33(12):1677–85. Available from: http://www3.interscience.wiley.com/journal/119818374/grouphome/home.html 10.1111/dme.13167 27279343

[15] Casey D, De Civita M, Dasgupta K. Understanding physical activity facilitators and barriers during and following a supervised exercise programme in Type 2 diabetes: A qualitative study. Diabet Med [Internet]. 2010;27(1):79–84. Available from: http://ep.fjernadgang.kb.dk/login?url=http://ovidsp.ovid.com/ovidweb.cgi?T=JS&CSC=Y&NEWS=N&PAGE=fulltext&D=emed12&AN=358096460 10.1111/j.1464-5491.2009.02873.x 20121893

[16] Tulloch H, Sweet SN, Fortier M, Capstick G, Kenny GP, Sigal RJ. Exercise facilitators and barriers from adoption to maintenance in the diabetes aerobic and resistance exercise trial. Can J Diabetes. 2013/12/11. 2013;37(6):367–74. 10.1016/j.jcjd.2013.09.002 24321716

[17] Dave D, Soni S, Irani A. Identification of barriers for adherence to exercise in type 2 diabetes mellitus-a cross sectional observational study. Physiother (United Kingdom) [Internet]. 2015;101:eS297 Available from: http://ep.fjernadgang.kb.dk/login?url=http://ovidsp.ovid.com/ovidweb.cgi?T=JS&CSC=Y&NEWS=N&PAGE=fulltext&D=emed17&AN=72113987

[18] de Jongh T, Gurol-Urganci I, Vodopivec-Jamsek V, Car J, Atun R. Mobile phone messaging for facilitating self-management of long-term illnesses. Cochrane Database Syst Rev Database Syst Rev [Internet]. 2012;(12):CD007459.pub2. Available from: http://onlinelibrary.wiley.com/doi/10.1002/14651858.CD007459.pub2/pdf/standard 10.1002/14651858.CD007459.pub2 PMC6486189 23235644

[19] Russell-Minda E, Jutai J, Psych C, Speechley M, Bradley K, Sc BH. Health Technologies for Monitoring and Managing Diabetes : A Systematic Review. J Diabetes Sci Technol. 2009;3(6):1460–71. 10.1177/193229680900300628 20144402 PMC2787048

[20] Arambepola C, Ricci-Cabello I, Manikavasagam P, Roberts N, French DP, Farmer A. The impact of automated brief messages promoting lifestyle changes delivered via mobile devices to people with type 2 diabetes: A systematic literature review and meta-Analysis of controlled trials. J Med Internet Res. 2016;18(4):1–17. 10.2196/jmir.5425 PMC4873307 27095386

[21] Kongstad MB, Valentiner LS, Ried-Larsen M, Walker KC, Juhl CB, Langberg H. Effectiveness of remote feedback on physical activity in patients with type 2 diabetes: a systematic review and meta-analysis of randomized controlled trials. J Telemed Telecare. 2017;0(0):1–9. 10.1177/1357633X17733772 28958212

[22] Cassimatis M, Kavanagh DJ. Effects of type 2 diabetes behavioural telehealth interventions on glycaemic control and adherence: a systematic review. J Telemed Telecare. 2012/12/05. 2012;18(8):447–50. 10.1258/jtt.2012.GTH105 23209266

[23] Harrison S, Stadler M, Ismail K, Amiel S, Herrmann-Werner A. Are Patients with Diabetes Mellitus Satisfied with Technologies Used to Assist with Diabetes Management and Coping: A Structured Review. Diabetes Technol Ther [Internet]. 2014;16(11):771–83. Available from: http://online.liebertpub.com/doi/abs/10.1089/dia.2014.0062 25069057 10.1089/dia.2014.0062

[24] Schoeppe S, Alley S, Lippevelde W Van, Bray NA, Williams SL, Duncan MJ, et al Efficacy of interventions that use apps to improve diet, physical activity and sedentary behaviour : a systematic review. Int J Behav Nutr Phys Act [Internet]. 2016;13(27):1–26. Available from: 10.1186/s12966-016-0454-y 27927218 PMC5142356

[25] Connelly J, Kirk A, Masthoff J, MacRury S. Systematic Review or Meta-analysis The use of technology to promote physical activity in Type 2 diabetes management : a systematic review. DiabeticMedicine. 2013;30:1420–32. 10.1111/dme.12289 23870009

[26] Orr JA, King RJ. Mobile phone SMS messages can enhance healthy behaviour: a meta-analysis of randomised controlled trials. Health Psychol Rev. 2015;9(4):397–416. 10.1080/17437199.2015.1022847 25739668

[27] Tamban CA, Isip-Tan IT, Jimeno CA. A randomized controlled trial on the use of short message services for improving adherence to diet and exercise among patients with type 2 diabetes mellitus at the university of the Philippines-Philippine general hospital diabetes clinic. Endocr Rev [Internet]. 2013;34(3 SUPPL. 1):1–20. Available from: http://press.endocrine.org/doi/abs/10.1210/endo-meetings.2013.DGM.6.SAT-80122947396

[28] Agboola S, Jethwani K, Lopez L, Searl M, O’Keefe S, Kvedar J. Text to move: A randomized controlled trial of a text-messaging program to improve physical activity behaviors in patients with type 2 diabetes mellitus. J Med Internet Res. 2016;18(11):1–22. 10.2196/jmir.6439 PMC5135731 27864165

[29] Swoboda CM, Miller CK, Wills CE. Impact of a goal setting and decision support telephone coaching intervention on diet, psychosocial, and decision outcomes among people with type 2 diabetes. Patient Educ Couns [Internet]. 2017;100(7):1367–73. Available from: 10.1016/j.pec.2017.02.007 28215827

[30] Siminerio LM. Approaches to Help People With Diabetes Overcome Barriers for Improved Health Outcomes. Diabetes Educ. 2008;34(February):18–24. 10.1177/0145721707313938 18268002

[31] Ried-larsen M, Thomsen RW, Berencsi K, Brinkløv CF, Brøns C, Valentiner LS, et al Implementation of interval walking training in patients with type 2 diabetes in Denmark : rationale, design, and baseline characteristics. Clincal Epidemiol [Internet]. 2016;8:201–9. Available from: https://www.ncbi.nlm.nih.gov/pmc/articles/PMC4908935/pdf/clep-8-201.pdf 10.2147/CLEP.S97303 PMC4908935 27354828

[32] Eldridge SM, Lancaster GA, Campbell MJ, Thabane L, Hopewell S, Coleman CL, et al Defining feasibility and pilot studies in preparation for randomised controlled trials: Development of a conceptual framework. PLoS One. 2016;11(3):1–22. 10.1371/journal.pone.0150205 PMC4792418 26978655

[33] World Medical Association. World Medical Association Declaration of Helsinki: Ethical Principles for Medical Research Involving Human Subjects. Clin Rev. 2013;6–9. 10.1001/jama.2013.281053 24141714

[34] Eldridge SM, Chan CL, Campbell MJ, Bond CM, Hopewell S. CONSORT 2010 statement : extension to randomised pilot and feasibility trials The Consolidated Standards of Reporting Trials (CONSORT) statement reporting of randomised controlled an extension to that statement for. BMJ Br Med J. 2016;355:1–29.

[35] Brinkløv CF, Thorsen IK, Karstoft K, Brøns C, Valentiner L, Langberg H, et al v. BMC Sports Sci Med Rehabil [Internet]. 2016;8(1):31 Available from: http://bmcsportsscimedrehabil.biomedcentral.com/articles/10.1186/s13102-016-0056-7 28174664 10.1186/s13102-016-0056-7 PMC5290632

[36] Karstoft K, Brinkløv C, Thorsen I, N JS, Ried-Larsen M. Resting Metabolic Rate Does Not Change in Response to Different Types of Training in Subjects with Type 2 Diabetes. Clin Trial. 2017;8(June):1–10. 10.3389/fendo.2017.00132 PMC5468455 28659869

[37] Pedersen BK, Saltin B. Exercise as medicine—Evidence for prescribing exercise as therapy in 26 different chronic diseases. Scand J Med Sci Sport. 2015;25:1–72. 10.1111/sms.12581 26606383

[38] Miller WR. Rollnick S. Motivational Interviewing: Preparing People for Change. 2nd New York, NY: Guilford Press; 2002.

[39] Locke EA, Latham GP. New Directions in Goal-Setting Theory. SAGE. 2006;15(5):265–8.

[40] Locke EA, Latham GP. Building a Practically Useful Theory of Goal Setting and Task Motivation, A 35-Year Odyssey. Am Ps. 2002;57(9):705–17. 10.1037//0003-066x.57.9.705 12237980

[41] Portney LG, Watkins MP. Surveys and questionnaires In: Cohen M, Kerian M, editors. Foundations of Clinical Research—Applications to Practice. third edit Pearson International Edition; 2009 p. 335–57.

[42] Besson H, Brage S, Jakes RW, Ekelund U, Wareham NJ. Estimating physical activity energy expenditure, sedentary time, and physical activity intensity by self-report in adults. Am J Clin Nutr [Internet]. 2010;91(1):106–14. Available from: http://www.ajcn.org/cgi/content/abstract/91/1/106%5Cnhttp://ajcn.nutrition.org/content/91/1/106.short 10.3945/ajcn.2009.28432 19889820

[43] Ekelund U, Brage S, Franks PW, Hennings S, Emms S, Wareham NJ. Physical activity energy expenditure predicts progression toward the metabolic syndrome independently of aerobic fitness in middle-aged healthy caucasians: The medical research council ely study. Diabetes Care. 2005;28(5):1195–200. 15855588 10.2337/diacare.28.5.1195

[44] Ware John E, Kosinski Mark K S. A 12-Item Short-Form Health Survey: COnstruction of Scales and Preliminary Tests of Reliability and Validity. Med Care. 1996;34(3):220–33. 8628042 10.1097/00005650-199603000-00003

[45] Karstoft K, Winding K, Knudsen SH, Nielsen JS, Thomsen C, Pedersen BK, et al The Effects of Free-Living Interval- Walking Training on Glycemic Control, Body Composition, and Physical Fitness in Type 2 Diabetic Patients. Diabetes Care [Internet]. 2013;36(July 2012):228–36. Available from: http://www.ncbi.nlm.nih.gov/pubmed/23002086 23002086 10.2337/dc12-0658 PMC3554285

[46] Yom-Tov E, Feraru G, Kozdoba M, Mannor S, Tennenholtz M, Hochberg I. Encouraging Physical Activity in Patients With Diabetes: Intervention Using a Reinforcement Learning System. J Med Internet Res [Internet]. 2017;19(10):e338, p1–12. Available from: http://www.jmir.org/2017/10/e338/ 10.2196/jmir.7994 29017988 PMC5654735

[47] Morton K, Sutton S, Hardeman W, Troughton J, Yates T, Griffin S, et al A Text-Messaging and Pedometer Program to Promote Physical Activity in People at High Risk of Type 2 Diabetes: The Development of the PROPELS Follow-On Support Program. JMIR Mhealth Uhealth. 2015/12/19. 2015;3(4):e105 10.2196/mhealth.5026 26678750 PMC4704921

[48] Waki K, Aizawa K, Kato S, Fujita H, Lee H, Kobayashi H, et al DialBetics With a Multimedia Food Recording Tool, FoodLog: Smartphone-Based Self-Management for Type 2 Diabetes. J Diabetes Sci Technol. 2015/04/18. 2015;9(3):534–40. 10.1177/1932296815579690 25883164 PMC4604534

[49] Chlebowy DDO, Hood S, LaJoie AS. Facilitators and Barriers to Self-management of Type 2 Diabetes Among Urban African American Adults Focus Group Findings. Diabetes Educ [Internet]. 2010;36(6):897–905. Available from: http://www.ncbi.nlm.nih.gov/pubmed/20974906 10.1177/0145721710385579 20974906

[50] Polonsky WH, Fisher L. When Does Personalized Feedback Make A Difference? A Narrative Review of Recent Findings and Their Implications for Promoting Better Diabetes Self-Care. Curr Diab Rep. 2015;15(50):1–10. 10.1007/s11892-015-0620-7 26077015

[51] Michie S, Abraham C, Whittington C, Mcateer J. Effective Techniques in Healthy Eating and Physical Activity Interventions : A Meta-Regression. Heal Psychol. 2009;28(6):690–701. 10.1037/a0016136 19916637

[52] Yavuz DG, Bilen H, Sancak S, Garip T, Hekimsoy Z, Sahin I, et al Impact of telephonic interviews on persistence and daily adherence to insulin treatment in insulin-naïve type 2 diabetes patients: Dropout study. Patient Prefer Adherence. 2016;10:851–61. 10.2147/PPA.S100626 27274207 PMC4876103

[53] Ruck JM, Zhou S, Thomas AG, Segev DL, Cramm SL, Massie AB, et al Electronic messaging and communication with living kidney donors. Clin Transplant. 2017;(12):1–6. 10.1111/ctr.13184 PMC6116553 29281129

[54] Kebede M, Zeleke A, Asemahagn M, Fritz F. Willingness to receive text message medication reminders among patients on antiretroviral treatment in North West Ethiopia : A cross-sectional study. BMC Med Inform Decis Mak [Internet]. 2015;15(65):1–10. Available from: 10.1186/s12911-015-0193-z 26268394 PMC4535252

[55] Ainsworth J, Palmier-Claus JE, Machin M, Barrowclough C, Dunn G, Rogers A, et al A Comparison of Two Delivery Modalities of a Mobile Phone—Based Assessment for Serious Mental Illness : Native Smartphone Application vs Text—Messaging Only Implementations. J Med Internet Res. 2013;15(4):1–23. 10.2196/jmir.2328 PMC3636800 23563184

[56] Worawong C, Borden MJ, Cooper KM, Pérez OA, Lauver D. Evaluation of a Person-Centered, Theory-Based Intervention to Promote Health Behaviors. Nurs Res [Internet]. 2018;67(1):6–15. Available from: http://insights.ovid.com/crossref?an=00006199-201801000-00003 10.1097/NNR.0000000000000254 29240655 PMC9502240

[57] Dobson R, Carter K, Cutfield R, Hulme A, Hulme R, McNamara C, et al Diabetes Text-Message Self-Management Support Program (SMS4BG): A Pilot Study. JMIR Mhealth Uhealth. 2015/04/02. 2015;3(1):e32 10.2196/mhealth.3988 25830952 PMC4390615

[58] Horner GN, Agboola S, Jethwani K, Tan-McGrory A, Lopez L. Designing Patient-Centered Text Messaging Interventions for Increasing Physical Activity Among Participants With Type 2 Diabetes: Qualitative Results From the Text to Move Intervention. JMIR mHealth uHealth [Internet]. 2017;5(4):e54, p1–12. Available from: http://mhealth.jmir.org/2017/4/e54/ 10.2196/mhealth.6666 28438728 PMC5422654

[59] Ryan Rm, Williams GC, Patrick H, Deci E. Ryan Self-determination theory and physical activity. The dynamics of motivation and wellness (1).pdf. Hell J Psychol. 2009;6:107–24.

[60] Deci EL, Ryan RM. Facilitating optimal motivation and psychological well-being across life’s domains. Can Psychol Can [Internet]. 2008;49(1):14–23. Available from: http://psycnet.apa.org/journals/cap/49/1/14.pdf%5Cnhttp://search.proquest.com.ezproxy.library.ubc.ca/docview/220818810?accountid=14656%5Cnhttp://gw2jh3xr2c.search.serialssolutions.com/?ctx_ver=Z39.88-2004&ctx_enc=info:ofi/enc:UTF-8&rfr_id=info:sid/ProQ:cb

[61] Kinnafick FE, Thøgersen-Ntoumani C, Duda J. The effect of need supportive text messages on motivation and physical activity behaviour. J Behav Med. 2016;39(4):574–86. 10.1007/s10865-016-9722-1 26915963 PMC4942483

[62] Karstoft K, Clark MA, Jakobsen I, Müller IA, Pedersen BK, Solomon TPJ, et al The effects of 2 weeks of interval vs continuous walking training on glycaemic control and whole-body oxidative stress in individuals with type 2 diabetes: a controlled, randomised, crossover trial. Diabetologia. 2017;60(3):508–17. 10.1007/s00125-016-4170-6 27942800

[63] Masuki S, Morikawa M, Nose H. Interval Walking Training Can Increase Physical Fitness in Middle-Aged and Older People. Exerc Sport Sci Rev. 2017;45(3):154–62. 10.1249/JES.0000000000000113 28418999

[64] Nemoto K, Gen-no H, Masuki S, Okazaki K, Nose H. Effects of high-intensity interval walking training on physical fitness and blood pressure in middle-aged and older people. Mayo Clin Proc [Internet]. 2007;82(7):803–11. Available from: http://ovidsp.ovid.com/ovidweb.cgi?T=JS&CSC=Y&NEWS=N&PAGE=fulltext&D=med5&AN=17605959%5Cnhttp://linksource.ebsco.com/ls.7f1c15d4-0de3-41c4-b53c-cda34973d688.true/linking.aspx?sid=OVID:medline&id=pmid:17605959&id=doi:&issn=0025-6196&isbn=&volume=82&issue=7 17605959 10.4065/82.7.803

[65] Morikawa M, Okazaki K, Masuki S, Kamijo Y, Yamazaki T, Gen-no H, et al Physical fitness and indices of lifestyle-related diseases before and after interval walking training in middle-aged and older males and females. Br J Sports Med. 2011;45(3):216–24. 10.1136/bjsm.2009.064816 19846423

[66] Boulé NG, Haddad E, Kenny GP, Wells G a, Sigal RJ. Effects of exercise on glycemic control and body mass in type 2 diabetes mellitus: a meta-analysis of controlled clinical trials. JAMA. 2001;286(10):1218–27. 11559268 10.1001/jama.286.10.1218

[67] Cavanaugh KL. Health literacy in diabetes care: explanation, evidence and equipment. Diabetes Manag. 2011;1(2):191–9. 10.2217/dmt.11.5 PMC3158575 21860659

[68] Muller I, Stuart B, Hayter V, Little P, Ganahl K, Doyle G, et al Effects on Engagement and Health Literacy Outcomes of Web—Based Materials Promoting Physical Activity in People With Diabetes : An International Randomized Trial. J Med Internet Res. 2017;19(1):1–21. 10.2196/jmir.6601 PMC5294369 28115299

[69] Powell CK, Hill EG, Clancy DE. The Relationship Between Health Literacy and Diabetes Knowledge and Readiness to Take Health Actions. Diabetes Educ. 2007;33(1):144–51. 10.1177/0145721706297452 17272800

[70] Chahardah-cherik S, Gheibizadeh M, Jahani S, Cheraghian B. The Relationship between Health Literacy and Health Promoting Behaviors in Patients with Type 2 Diabetes. IJCBNM. 2018;6(1):65–75. 29344537 PMC5747574

[71] Caruso R, Magon A, Baroni I, Dellafiore F. Health literacy in type 2 diabetes patients : a systematic review of systematic reviews. Acta Diabetol. 2018;55(1):1–12. 10.1007/s00592-017-1071-1 29129000

[72] Leung L, Cheng C. e-Helth/m-Health Adoption and Lifestyle Improvements: Exploring the Roles of Tecnology Readiness, the Expectation-Confimation Model, and Health-Related Information Activities. 14th Int Telecommun Soc Asis-Pacific Reg Conf. 2017;1–37.

[73] Shiyanbola OO, Unni E, Huang Y, Lanier C. The association of health literacy with illness perceptions, medication beliefs, and medication adherence among individuals with type 2 diabetes. Res Soc Adm Pharm [Internet]. 2017;IN PRESS(8):1–7. Available from: 10.1016/j.sapharm.2017.12.005 29317189

[74] Schillinger D, Grumbach K, Piette J, Wang F, Osmond D, Daher C, et al Association of Health Literacy With Diabetes Outcomes. JAMA—J Am Med Assoc. 2002;288(4):475–82. 10.1001/jama.288.4.475 12132978

[75] Bergner EM, Nelson LA, Rothman RL, Mayberry L. Text Messaging May Engage and Benefit Adults with Type 2 Diabetes Regardless of Health Literacy Status. Heal Lit Res Pr. 2017;1(4):1–18. 10.3928/24748307-20170906-01 PMC5714586 29214241

[76] Edison SW, Geissler GL. Measuring attitudes towards general technology: Antecedents, hypotheses and scale development. J Targeting, Meas Anal Mark. 2003;12(2):137–56.

[77] Kayser L, Kushniruk A, Osborne RH, Norgaard O, Turner P. Enhancing the Effectiveness of Consumer-Focused Health Information Technology Systems Through eHealth Literacy : A Framework for Understanding Users ‘ Needs. JMIR Hum FACTORS. 2015;2(1):1–14. 10.2196/humanfactors.3696 PMC4797661 27025228

[78] Prince SA, Adamo KB, Hamel ME, Hardt J, Gorber SC, Tremblay M. A comparison of direct versus self-report measures for assessing physical activity in adults : a systematic review. Int J Behav Nutr Phys Act. 2008;5(56):1–24. 18990237 10.1186/1479-5868-5-56 PMC2588639

[79] Runz-jørgensen SM, Schiøtz ML, Christensen U. Perceived value of eHealth among ple living with multimorbidity : a qualitative study. J Comorbidity. 2017;7(1):96–111. 10.15256/joc.2017.7.98 PMC5777537 29359124

