# Supplementary material for: Effect of ecological momentary assessment, goal-setting and personalized phone-calls on adherence to interval walking training using the InterWalk application among patients with type 2 diabetes—A pilot randomized controlled trial
Source: PLoS One. 2019 Jan 10;14(1):e0208181. doi: 10.1371/journal.pone.0208181 (PMC6328102; doi:10.1371/journal.pone.0208181)
Supplement: S1 File — (PDF) [file pone.0208181.s001.pdf]

Protokol-40330_v3_2014

Title: The smart-phone as a physical fitness monitor on a population level – validity and sensitivity

BACKGROUND

Low physical fitness is an independent predictor of a range of non-communicable diseases including Type 2 Diabetes (T2D) and all-cause mortality [1-3]. In addition, peak oxygen consumption (VO_{2peak}) is the most validated marker of cardiopulmonary exercise capacity, which is used in daily clinic for investigating cardiac and pulmonary disorders [4, 5]. Valid continuous monitoring of VO_{2peak} would thus be of clinical and scientific importance.

To improve VO_{2peak} and physical activity in T2D patients we recently examined the feasibility of implementing Interval walking (IWT) and continuous walking training in a sample of T2D patients in a four-month trial [6]. A small tri-axial accelerometer training device (JD Mate; Kissei Comtec, Matsumoto, Japan) was employed to initiate and monitor exercise. IWT, but not continuous walking, resulted in remarkably beneficial effects on physical fitness level (16%). Moreover, this was achieved with a compliance rate of ~90% and low drop-out. Thus the exercise modality and the device employing it seem very feasible. The device individualizes exercise intensity by using the onboard accelerometer to estimate VO_{2peak}. This has been validated in an elderly Japanese sample [7], but it remains unknown to what extent estimated VO_{2peak} is sensitive to exercise adaptation, therefore unknown if it can be used in clinic and interventional research. Due to the large costs (2.500 Danish kr.) and lack of stability of the training device, implementation of IWT and the fitness test in clinic and in larger samples is difficult and expensive. Therefore, we developed the free InterWalk smart-phone application as an interventional tool for the 'Danish Centre for Strategic Research in Type 2 Diabetes' (DD2). Briefly, the DD2 aims is to prevent co-morbidities and to ensure normal quality of life and life expectancy in newly diagnosed T2D patients through range of strategies, among these are increases in exercise and physical activity [8].

The application is a free personal trainer individualizing IWT by employing the on-board accelerometer and global positioning system (GPS) to specify walking intensity and speed. Intensities are determined, as with the accelerometer-based training device with the option of including GPS determined walking speed. Data from the fitness test is logged on a server, and as the CPR-number is a part of the profile information on the application, it will enable researchers and clinicians to combine it with health information using the Danish registers. However, the validity of the VO_{2peak} test functions and sensitivity to exercise adaptation of smart-phone based tests are at large, unknown. As the DD2 are establishing a large cohort consisting of ~50.000 newly diagnosed T2D patients, a valid tool for assessing exercise capacity on a large scale is needed [8]. Moreover, if valid, this application provides clinicians and researchers with an easy and cheap tool to estimate physical fitness in clinic and in very large samples and not only in T2D patients.

The VO_{2peak} test is conducted by the patients using their smart-phones and it is based on three self-selected walking paces - slow, moderate and high. It is uncertain to which extent the self-selected walking paces are adequate to reflect a VO_{2peak}. Furthermore, as T2D patients have limited experience with exercise training, we speculate that acclimatization to high intense walking and pacing during the $VO2_{peak}$ test can improve the validity.

The aim of the study is to validate the ability of the smart-phone based IWT application to accurately reflect physical fitness in a sample of T2D patients. More specifically we aim to;

1) Develop an algorithm for conversion of the VO_{2peak} test output to energy expenditure and validate the algorithm against maximal oxygen consumption in an independent sample. We hypothesize that the VO_{2peak} test algorithm will be a valid measure of physical fitness if the peak test is performed at a sufficient walking pace.
2) Investigate the effect of acclimatization to high speed walking on the ability of the patient to perform a valid VO_{2peak} test. We hypothesize that acclimatization will increase walking economy in

the patients thus increase the walking pace during the VO_{2peak} test without any increase in physical fitness.

3) To investigate sensitivity of the VO_{2peak} test function to reflect actual changes in the maximal oxygen consumption. We hypothesize that a three-month IWT intervention will improve the maximal oxygen consumption and the improvement will be reflected in the VO_{2peak} test.

STUDY DESIGN

Subjects, screening and sample size: A total of n=50 TD2 patients will be included in the study. All patients will undergo the protocol and intervention. However, 25 persons will be randomly chosen to act as a cross-validation reference group. Their data will act as reference to the developed algorithm. Based on previous studies on the association between VO_{2peak} and the maximal oxygen consumption (VO_{2max}) we expect a correlation of 0.7. With a power of 0.8 (1-β) and α set at 0.05, allowing for four covariates we need a least 23 subjects undergo the study (group 1). With an *a priori* difference of 0.1 between the correlation coefficient (ρ^2) in group 1 and the correlation coefficient in the independent sample (group 2) (ω^2), allowing for four covariates with we need 25 subjects in the cross-validation sample [9]. A medical examination, standard anthropometric measures, medical history, blood chemistry screen, ECG, oral glucose tolerance test (OGTT), Dual x-ray absorptiometry (DXA) scan, an assessment of walking economy, assessment of resting energy expenditure (REE), a smart-phone based VO_{2peak} test and a laboratory VO_{2max} test will be performed. If an individual meets the inclusion criteria, informed oral and written consent will be obtained prior to participation in the study, in accordance with guidelines developed by the ethical committees. For participants without a smart-phone the exercise and VO_{2peak} tests will administered on an IPod Touch.

Inclusion Criterias: T2D, age 30-70 y, BMI >18 but <40 kg/m2 and confirmed T2D diagnose.

Exclusion Criterias: Pregnancy, smoking, contraindication to increased levels of physical activity [10], insulin dependence, evidence of thyroid, liver, lung, heart or kidney disease

Recruitment, information and informed consent: Potential subjects are recruited through advertising on "www.forsogsperson.dk" and advertising in local health care centers. Potential subjects contact one of the project participants by telephone and are orally informed about the study. If no exclusion criteria are identified through the telephone interview, the written material and consent form is mailed to the participant with a free post envelope and he/she will be encouraged to read the information thoroughly. The potential participant is furthermore offered an information meeting, and the potential subject is informed about the possibility of inviting a private counselor to the information meeting. If the information meeting is requested, the study information will be given in a closed room where only the scientific participant, the potential subject and the potential private counselor will be present. If the potential subject is ready and wants to participate in the study, the study participant will be required to mail the informed consent form no earlier than 24 hours after the meeting. If the potential subject wants additional time to consider whether or not to participate in the study, an agreement about a telephone meeting after approx. one week is made. The subject will be informed of the possibility of contacting one of the scientific participants by telephone, in case of questions. If no additional oral information, besides the information provided initially by phone, is needed, the possible participant can return the consent form no earlier than 24 hours upon reception of the written material. Upon reception of the consent form by the scientific staff, the subject will be contacted by a member of the scientific staff and a date for the first visit is arranged.

Study design: The study is a validation study where the primary endpoint is $VO2_{peak}$ and indirect calorimetry is used as reference to assess face validity of the InterWalk test. Data from all the study participants will serve a material for development of algorithms to predict $VO2_{peak}$. Data from the first and second lab day will form basis for a cross validation. Data from the third lab day will serve as material to

detect the sensibility in changes in $VO2_{peak}$. Throughout the study period all participants will use the InterWalk application to engage in interval walking training (exposure) [6]. To assess convergent validity and to confirm diabetes status an OGTT is employed. Study participants will complete a variety of tests across three laboratory days, over a period of three months. Table 1 describes the sequence of the tests included in the study. The study timeline is depicted in Figure 1. Participants will be instructed to fast, refrain from smoking and physical activity for 9 hours prior to the tests. After the assessment or REE or the OGTT the participants will be offered a meal.

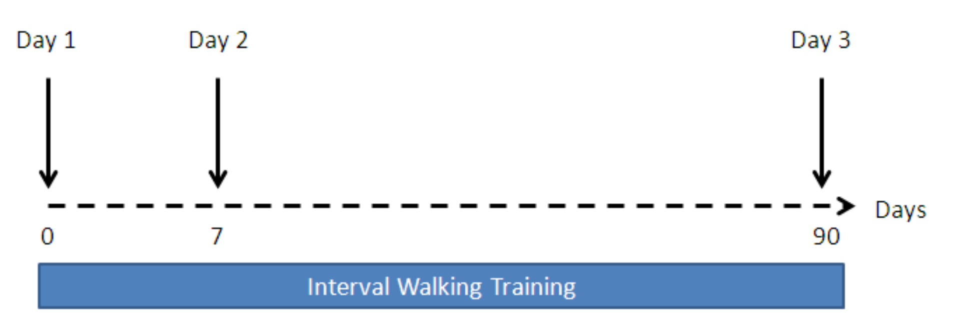

Figure 1: Study timeline

Table 1 Measurements included in the study

**Day 1**	**Day 2**	**Day 3**
Medical examination<br>Assessment of resting energy expenditure (REE)<br>Anthropometric measures<br>Self-administered free living smart-phone based VO2peak test<br>Paced free-living smart-phone based VO2peak test<br>Assessment of walking-economy<br>A graded VO2max test	REE<br>2-hour Oral glucose tolerance test (OGTT)<br>DXA scan<br>Self-administered Smart-phone based VO2peak test<br>Paced smart-phone based VO2peak test<br>Assessment of walking-economy	As day 2<br>A graded VO2max test

METHODS

Interval walking training: After day 1 the participants will engage in a three-month interval walking training program. An Interval-walking bout will consist of repeated cycles of 3 minutes of fast and 3 minutes of slow walking at 70% and 40% of the smart-phone assessed peak VO_2-rate, respectively. IWT is conducted for at least 60 minutes per day, three days per week. Compliance is monitored continuously using the on-board accelerometer logger.

Estimation of VO_{2max}: VO_{2max} will be assessed by employing a graded walking test protocol on a treadmill (Technogym Runrace, Gambettola, Italy). The test consists of a 5-min warm-up (individually determined moderate walking pace, 0% incline) followed by 2-min stages of increasing inclines (2% per stage) at individually determined brisk walking pace until the following criteria were met: plateauing of heart rate and VO_2 with incremental workloads, respiratory exchange ratio > 1.1 [6]. Oxygen consumption will be assessed using continuous indirect calorimetric measurements (CPET, Cosmed, Italy) and heart rate monitoring (Polar Electro, Holte, DK).

Walking Economy: To assess economy during fast walking, the steady-state oxygen consumption will be estimated during steady-state walking at a fixed speed on a treadmill. (Technogym Runrace, Gambettola, Italy). Measurements are made after a 15 minute customization period at 3 km/h. After the end of the period, measurements also are made over 5 minutes at the participants fastest self-selected pace to provide additional time for patients to reach steady-state oxygen consumption. The mean oxygen consumption of the later 2 minutes will be used. Oxygen consumption will be assessed using continuous indirect calorimetric measurements (CPET, Cosmed, Italy) and heart rate monitoring (Polar Electro, Holte, DK). The test will be performed at the same speed at all test days.

Demographic Information: Height, weight, waist and hip circumference will be measured by standard procedures. Dual x-ray absorptiometry (iDXA; Lunar, Madison, WI) will be used to assess whole body adiposity.

Smart-phone administered VO_{2peak} test: The test consists of four stages; 1) 30 seconds of standing still, 2) 2 minutes of slow walking, 3) 2 minutes of moderate walking at moderate intensity, 4) 2 minutes of walking at high intensity. The three later paces are self-selected. It is recommended that the phone is place at the hips. However, it is likely that the phone might be placed at other body locations. Therefore the participant will be fitted with three phones; 1) at the hip, 2) hand-held and 3) on their upper-arm. Throughout the tests oxygen consumption will be monitored using continuous indirect calorimetric measurements (CPET, Cosmed, Italy) and heart rate monitoring (Polar Electro, Holte, DK). The test will be conducted as both a self-administered test and where the participants are paced during walking. To ensure test-retest reliability of the self-administered test, the participants perform two additional tests at home with two and four days apart from the second test day.

Glucose Tolerance: Following an overnight fast, an antecubital intravenous (i.v.) line will be placed and fasting plasma glucose, insulin, C-peptide, lipids, cytokines, and HbA1c will be determined. A standard 75 g oral glucose tolerance test (OGTT) will be performed over 2 hours with blood draws every 30 min for the measurement of glucose, insulin, and C-peptide.

Resting Energy Expenditure (REE): REE will be assessed via indirect calorimetry (Quark B2, Cosmed, Italy). REE will be assessed using a ventilated hood as described earlier [11].

BIOLOGICAL MATERIAL
Blood will be collected in the study and stored in a research biobank throughout the study. This will be used to assess the potential differential changes in variables following the interventions. Approx. 100 mL blood will be collected per trial day. Total blood sampling will be approx. 300 mL. After the study termination the remaining biological material will anonymously get stored in a biobank for a maximum of 20 years and after that, it will be destroyed. If any later studies want to make use of the biological material, this will only take place following approval by the ethical committee. The biological material will not leave Denmark.

STATISTICAL ANALYSES
Based on previous research it is assumed that the smartphone accelerometer output increases linearly until app. 9-10 km/h [12]. As it is not expected that our participant exceed this speed, backward stepwise *linear* regression analysis will be employed to develop an appropriate algorithm. In the full model we will include age, height, gender and monitor placement as co-variates. Log-likelihood test of the model fit will be employed to find the best fit to the data. Standard assumptions, such as linearity, independence between co-variates and normality of residuals will be checked. Bland-Altman blots will be used to assess the validity of the algorithm in an independent sample.

FEASIBILITY
In collaboration with the DD2 IWT committee (Professor Bente Klarlund Pedersen, Professor Henning Langberg, and Professor, chief clinician Allan Vaag, Ph.D. fellow Kristian Karstoft, Post. Doc. Charlotte Brøns and DD2 project coordinator Jens Steen Nielsen) I have designed the protocol and results from the study will be implemented in the DD2. I have previously conducted validation studies on measurements of free-living physical activity and am an expert in measurements of physical fitness and metabolic monitoring [13-16]. The study is conducted in close collaboration with Dr. Kristian Karstoft who is an expert in the techniques necessary to complete glucose tolerance tests and DXA.

RISKS, SIDE EFFECTS AND DISCOMFORT
VO_2-max test: A physical fitness test, where subjects must put in maximum effort. This will cause some degree of breathlessness. VO_2max test is a standard method used for scientific purposes in our laboratory.

DXA scan: Is not expected to cause significant discomfort. The radiation acquired is so small that it does not pose any risk to subjects. DXA-scan is a standard method used for scientific purposes in our laboratory.

Blood sampling: Will cause minor discomfort in terms of a venous catheter. There is theoretically a risk for infections introduced through the catheter. The blood volume collected is so small that it will cause no symptoms. Blood sampling is a standard method used for scientific purposes in our laboratory.

Indirect calorimetry: This is performed in a ventilated hood where the subject has to lie under a transparent hood. People with serious claustrophobia may in this feel some discomfort. Indirect calorimetry is a standard method used for scientific purposes in our laboratory

EXPECTED OUTCOMES
It is expected that a smart-phone based peak-test can act as valid marker of VO_{2peak}. Due to the unique setup, where data is logged continuously and the ability to link with central registers, this will potentially enable researches to monitor the effect of interventions on a very large scale. If the test is easy, cheap and validated it is likely that the use of cardiopulmonary testing in would increase in clinic. We believe that the

importance of the findings is of value for the society and fully justify potential discomfort perceived by the participants.

ETHICAL CONSIDERATIONS

The project is expected to result in limited risks, adverse effects and discomfort to the subjects. The subjects will benefit from the study in terms of a thorough medical examination, physical capacity and in terms of introduction to effective training methods. The study is sound and important, and it will contribute to our knowledge about how to assess and thus increase public health in a large and growing cohort of people with T2D.

PROTECTION OF PERSONAL INFORMATION

A unique subject ID number will be ascribed TO all participants to anonymize data. Identification key (ID to personal information) will be encrypted and stored securely and separately from the unique ID number and database on the research centre server. All data will be backed up on a secure server on a daily basis. Only the study researchers (Mathias Ried-Larsen and Kristian Karstoft) will access data directly. The study will be reported to "Datatilsynet" through Rigshospitalets joint review. The "Lov om behandling af personoplysninger" will be respected.

COMPENSATION

All participants will receive 2.250 Dkr. to cover lost earnings, transport and discomfort. The transaction is completed upon completion of the study (all lab days or upon withdrawal). For every completed day of laboratory testing, participants will receive 750 Dkr.

FUNDING

The study was initiated by the Centre of Inflammation and Metabolism (Post Doc, Ph.D. Mathias Ried-Larsen). The study has received funding from the Danish Diabetes Academy supported by the Novo Nordic Foundation. The financial support covers salary (270.000 Dkr.) for responsible researcher. Remaining expenses (317.500 Dkr.) are covered by the Centre of Inflammation and Metabolism. The Researchers declare no conflicts of interest.

DISSEMINATION

At least one manuscript will be produced from the data and published in an international peer-reviewed journal. All results will be disseminated (negative, positive and inconclusive findings).

STUDY LOCATION

Centre of Inflammation and Metabolism (CIM)
Rigshospitalet
Tagensvej 20, section M7641
DK-2100 Copenhagen
Denmark

SCIENTIFIC PARTICIPANTS

Mathias Ried-Larsen (Responsible), Cand. Scient, Ph.D., Post doc, CIM
Kristian Karstoft, MD, Ph.D. fellow, CIM
Bente Klarlund Pedersen, MD, DMsc, professor, CIM

REFERENCES

1. Gulati, M., et al., *The prognostic value of a nomogram for exercise capacity in women.* N Engl J Med, 2005. **353**(5): p. 468-75.
2. Hainer, V., H. Toplak, and V. Stich, *Fat or fit: what is more important?* Diabetes Care, 2009. **32 Suppl 2**: p. S392-7.
3. Kodama, S., et al., *Cardiorespiratory fitness as a quantitative predictor of all-cause mortality and cardiovascular events in healthy men and women: a meta-analysis.* JAMA, 2009. **301**(19): p. 2024-35.
4. Arena, R., et al., *Assessment of functional capacity in clinical and research settings: a scientific statement from the American Heart Association Committee on Exercise, Rehabilitation, and Prevention of the Council on Clinical Cardiology and the Council on Cardiovascular Nursing.* Circulation, 2007. **116**(3): p. 329-43.
5. Gargiulo, P., et al., *Predicted values of exercise capacity in heart failure: where we are, where to go.* Heart Fail Rev, 2013.
6. Karstoft, K., et al., *The effects of free-living interval-walking training on glycemic control, body composition, and physical fitness in type 2 diabetic patients: a randomized, controlled trial.* Diabetes Care, 2013. **36**(2): p. 228-36.
7. Yamazaki, T., et al., *A new device to estimate VO2 during incline walking by accelerometry and barometry.* Med Sci Sports Exerc, 2009. **41**(12): p. 2213-9.
8. Steffensen, C., et al., *The Danish Centre for Strategic Research in Type 2 Diabetes (DD2) Project: rationale and planned nationwide studies of genetic predictors, physical exercise, and individualized pharmacological treatment.* Clin Epidemiol, 2012. **4**(Suppl 1): p. 7-13.
9. Algina, J.a.K., H. J., *Cross-Validation Sample Sizes.* Applied Psychological Measurement, 2000. **24**(2): p. 173-179.
10. Pedersen, B.K. and B. Saltin, *Evidence for prescribing exercise as therapy in chronic disease.* Scand J Med Sci Sports, 2006. **16 Suppl 1**: p. 3-63.
11. Frayn, K.N., *Calculation of substrate oxidation rates in vivo from gaseous exchange.* J Appl Physiol, 1983. **55**(2): p. 628-34.
12. Brage, S., et al., *Reexamination of validity and reliability of the CSA monitor in walking and running.* Med Sci Sports Exerc, 2003. **35**(8): p. 1447-54.
13. Grontved, A., et al., *Independent and Combined Association of Muscle Strength and Cardiorespiratory Fitness in Youth With Insulin Resistance and beta-Cell Function in Young Adulthood: The European Youth Heart Study.* Diabetes Care, 2013. **36**(9): p. 2575-81.
14. Moller, N.C., et al., *Cardiovascular disease risk factors and blood pressure response during exercise in healthy children and adolescents: the European Youth Heart Study.* J Appl Physiol, 2010. **109**(4): p. 1125-32.
15. Ried-Larsen, M., et al., *Mechanical and free living comparisons of four generations of the Actigraph activity monitor.* Int J Behav Nutr Phys Act, 2012. **9**: p. 113.
16. Ried-Larsen, M., et al., *Physical activity intensity and subclinical atherosclerosis in Danish adolescents: the European Youth Heart Study.* Scand J Med Sci Sports, 2013. **23**(3): p. e168-77.

Additional Protocol 1
Project title: The smartphone as a physical fitness monitor on a population level – validity and sensitivity.
Change: Inklusionskriterie angående øvrig aldersgrænse
Argumentation for change: Vi har erfaret at en stor de laf vores målgruppe er ældre end 70år.
Ethical considerations: Vi har tidligere haft forsøgspersoner med kronisk sygdom, som diabetes type 2, som deltagere I træningsforsøg med højere intensitet (endda med intervalgang) uden sideeffekter (eks. Karstoft et al 2013, Diabetes Care). Da alle deltagere screenes for kontraindikationer mod fysisk aerob træning a fen læge inden inklusion I studiet og trænings-intensitet og volumen ikke overstiger de gældende anbefalinger fra Sundhedsstyrelsen, finder vi det etisk forsvarligt at personer over 70år kan deltage I studiet.

Additional Protocol 2

4) A secondary aim of the study is to explore if support to patients doing interval walking with the InterWalk app influence exercise adherence.

All patients are randomized using random permuted blocks (2x2) and stratification by sex in the two groups across the allocation period. One group will receive support and one group will act as control group during the intervention. The support group will in addition to the intervention be encouraged to set individual goals during the intervention, receive short message service (SMS) and be given the opportunity to walk in a group. Patients in the control group receive the intervention and no other support. All patients can participate in other offers from the municipality. Exercise adherence is measured by the exercise log in the InterWalk in minutes per week and secondary in numbers of exercise sessions per week.

The support options:

a) Individual Goal Setting: 2-3 functional goals related to everyday life evaluated every 4th week by phone.

b) SMS sent every Sunday with the question: "How many times have you done Interval walking using the InterWalk during the past week?". There will be 5 possible responses, 1. I have walked more than 3 times, 2. I have walked 3 times, 3. I have walked 2 times, 4. I have walked 1 time, 5. I have not walked at all. If the patient answers 1 or 2 a new SMS is sent with the text ”Good job, keep on doing interval walking". If the patient answers 3 or 4 a SMS with the text: "Good job, keep on doing interval walking. If you can make room for more than 1 or 2 passes of interval walking during the week it would be very good". If the patient answers option number 5, the patient will receive a SMS with the text: ”Remember that all physical activity in everyday life is important. Please specify the reason for not walking" with 7 possible responses: 1. The InterWalk app teases me, 2. I have been sick, 3. I don't feel like walking, 4. I don't want to walk alone, 5. Bad weather, 6 Too much work 7. Other cause. You can give more than one answer. All responses will cause a telephone call from a project member about motivation and barriers for interval walking.

c) Interval walk in a group: A project member will arrange group walking continuously during the intervention, if the patients have the need.

REASONS FOR CHANGE

This trial gives insights into facilitating and motivating factors or barriers of importance for physical activity in everyday life among the participating patients with type 2 diabetes. Additional supportive tools i.e. individual goal setting, SMS-track, telephone calls and the option of performing interval walking in a group provide patients with the possibility of giving direct feedback to the project leader.. The aim is to overcome some of the barriers for adherence to physical activity in patients with type 2 diabetes and ultimately to increase the benefits from the training. Results from this trial will provide a qualified answer to which support tools may be used in other projects where the aim is increase individual motivation and lower barriers for physical activity.

Randomisation of patients is necessary as this methodological approach allows for comparing the two groups in the trial. Results from this trial will be considered and applied in the design of a randomised controlled trial with 3 groups, where the InterWalk app will be used and where one group, in addition to the InterWalk app, will receive motivational support during the intervention period. Participating patients may withdraw from the trial at any time without consequences and influence on their other treatment.

None of the additional supportive tools (individual goal setting, SMS-track, telephone calls from project leader and possibility of interval walking in a group) that are part of the trial are associated with any risk or side effects. The participant information has been changed and all participating patients are informed of all aspects of the trial before considering participation.

REFERENCER

1. Avery L, Flynn D, van Wersch A, Sniehotta FF, Trenell MI. Changing physical activity behavior in type 2 diabetes: a systematic review and meta-analysis of behavioral interventions. Diabetes Care, 2012. 35(12):2681-9
2. Carlisle K, Warren R. A qualitative case study of telehealth for in-home monitoring to support the management of type 2 diabetes. Journal of Telemedicine and Telecare 2013:19(7):372-75
3. Eakin EG, Reeves MM, Lawler SP, Oldenburg B, Del Mar C, Wilkie K, Spencer A, Battistutta D, Graves N. The Logan Helathy Living Program: A cluster randomized trial of a telephone-delivered physical activity and dietary behavior intervention for primary care patients with type 2 diabetes or hypertension from a socially dis advantaged community – Rationale, design and recruitment. Comtemporary Clinical Trials 2008:29:439-45
4. Tatara N, Årsand E, Bratteteig T, Hartvigsen G. Usage and Perceptions of Mobile Self-Management Application for People with Type 2 Diabetes: Qualitative Study of a Five-Month Trial. IMIA and IOS Press 2013. Doi:10.3233/978-1-61499-289-9-127
